# Cholinergic modulation of striatal microcircuits

**DOI:** 10.1111/ejn.13949

**Published:** 2018-11-29

**Authors:** Nilupaer Abudukeyoumu, Teresa Hernandez‐Flores, Marianela Garcia‐Munoz, Gordon W. Arbuthnott

**Affiliations:** ^1^ Okinawa Institute of Science and Technology Graduate University Okinawa Japan

**Keywords:** acetylcholine, cholinergic interneurons, neuromodulation, striatum

## Abstract

The purpose of this review is to bridge the gap between earlier literature on striatal cholinergic interneurons and mechanisms of microcircuit interaction demonstrated with the use of newly available tools. It is well known that the main source of the high level of acetylcholine in the striatum, compared to other brain regions, is the cholinergic interneurons. These interneurons provide an extensive local innervation that suggests they may be a key modulator of striatal microcircuits. Supporting this idea requires the consideration of functional properties of these interneurons, their influence on medium spiny neurons, other interneurons, and interactions with other synaptic regulators. Here, we underline the effects of intrastriatal and extrastriatal afferents onto cholinergic interneurons and discuss the activation of pre‐ and postsynaptic muscarinic and nicotinic receptors that participate in the modulation of intrastriatal neuronal interactions. We further address recent findings about corelease of other transmitters in cholinergic interneurons and actions of these interneurons in striosome and matrix compartments. In addition, we summarize recent evidence on acetylcholine‐mediated striatal synaptic plasticity and propose roles for cholinergic interneurons in normal striatal physiology. A short examination of their role in neurological disorders such as Parkinson's, Huntington's, and Tourette's pathologies and dystonia is also included.

## Introduction

Cholinergic interneurons (ChIs) contribute to give striatum its place among structures with the highest levels of acetylcholine (ACh) in the brain (Zhou *et* *al.,* 2002). Without a doubt, these interneurons exert a strong and complex modulation of striatal microcircuits. These large interneurons form synapses with medium size spiny neurons (MSNs) and other numerous smaller GABAergic interneurons of which there are 10 subtypes and counting (Tepper & Koos *et* *al.,* 2017). ChIs can be identified by their electrophysiological characteristics (Goldberg & Wilson *et* *al.,* 2017) and by immunoreactivity of their enzymatic profile (Mesulam *et* *al.,* 1984). The morphology of ChIs, the richness of their synaptic contacts as well as the expression of a variety of receptors has attracted the attention of neuroscientists. More than 1000 research articles on ChIs, published during the last two decades, have enriched the understanding of their function.

## Striatal acetylcholine receptors

An early study indicated that destroying possible afferent pathways to striatum ‘cortex, thalamus, globus pallidus or ventrotegmental area’ did not affect the activity of choline acetylase nor acetylcholinesterase (AChE) or the histochemical staining within the nucleus (McGeer *et* *al.,* 1971; Lynch *et* *al.,* 1972). This led to the proposal that interneurons were the main intrinsic source of striatal ACh. We now know of external sources of ACh that arrives from the pedunculopontine and laterodorsal tegmental nuclei (Dautan *et* *al.,* 2014), but the main source of striatal ACh still is the spontaneously active ChIs (Kitai & Surmeier, 1993; Pisani *et* *al.,* 2007; English *et* *al.,* 2012; Goldberg *et* *al.,* 2012). At the cellular level, ACh exerts its actions through the activation of two families of receptors, muscarinic (mAChR) and nicotinic (nAChR). The mAChRs belong to the G‐protein‐coupled receptor (GPCR) family (Caulfield, 1993). These receptors are divided into group I (M_1_, M_3_, and M_5_) and group II (M_2_ and M_4_). Group I receptors are coupled to G_q/11_ proteins via α subunits that activate protein kinase C (PKC) and phospholipase C (PLC) leading to the production of inositol triphosphate and diacylglycerol that results in an increase in intracellular calcium. Group I receptors are found in striatal MSNs of both the direct (dMSN) and indirect (iMSN) pathways. In MSNs, these receptors are postsynaptically in dendritic spine necks and extrasynaptically locations (Hersch & Levey, 1995; Yan *et* *al.,* 2001). Group II receptors are coupled to G_i/o_ proteins, inhibit adenyl cyclase (AC) activity and close voltage‐activated calcium (Ca_v_) Ca_V_2 channels while opening inwardly rectifying potassium channels (Kir3) following GPCR activation (Caulfield, 1993; Nathanson, 2000; Eglen, 2006; Haga, 2013). Muscarinic M_2_ receptors act as autoreceptors on ChIs and are located mostly extrasynaptically suggesting a role in volume neurotransmission (Bernard *et* *al.,* 1998). M_2_ receptors act as inhibitory heteroreceptors on striatal neuropeptide Y‐somatostatin expressing (NPY‐SOM) GABAergic interneurons and on corticostriatal glutamatergic terminals (Hersch *et* *al.,* 1994; Bernard *et* *al.,* 1998).

The high degree of similarity of the orthosteric ligand‐binding site in all five types of muscarinic receptors is the main reason it has been difficult to identify subtype‐selective ligands (Eglen, 2006; Dencker *et* *al.,* 2012) and a reason why the dissection of specific cholinergic effects on neuronal activity and release has been difficult to achieve. Nevertheless, new pharmacological tools such as the highly specific antagonist peptide isolated from the green mamba snake venom are now being used (Jerusalinsky *et* *al.,* 2000; Karlsson *et* *al.,* 2000; Rowan & Harvey, 2011; Servent *et* *al.,* 2011). Similarly, positive allosteric modulators and allosteric agonists are becoming promising tools, even providing some therapeutic potential for several central nervous system diseases (Digby *et* *al.,* 2010; Bock *et* *al.,* 2017).

Acetylcholine release is regulated by presynaptically located hetero‐ and autoreceptors. Muscarinic autoreceptors M_2_/M_4_ (Hersch *et* *al.,* 1994; Ding *et* *al.,* 2006), via direct G_i/o_‐mediated inhibition of presynaptic Ca_V_2.2 and Ca_V_2.1 channels linked to exocytosis. Another presynaptic control of release is regulated by the M_4_ auto‐ and heteroreceptor activation of the barium‐sensitive potassium currents carried through K_ir_3 potassium channels in ChIs (Yan & Surmeier, 1996; Ding *et* *al.,* 2006) and corticostriatal terminals (Calabresi *et* *al.,* 1998a).

Nicotinic (nAChR) receptors are pentameric ligand‐gated ion channels that consist of either heteromeric subunit combinations of α subunits (α2‐10) and β subunits (β2‐4; Exley & Cragg, 2008; Gotti *et* *al.,* 2009). The most common types of nAChR in striatum are the homomeric α subunits (α7) and α4β2*. The α4β2* subcomposition acts as an autoreceptor in ChIs, as a postsynaptic heteroreceptor in GABAergic interneurons and as a presynaptic heteroreceptor in GABA, serotonin, and dopamine axon terminals (Eskow Jaunarajs *et* *al.,* 2015). The reported subunit composition on GABAergic interneurons is proposed to have the α4β2* and α4α5β2* subtypes (Eskow Jaunarajs *et* *al.,* 2015).

## Characteristics of cholinergic interneurons

### Anatomical

In general, anatomical studies have revealed that ChIs immunoreactive for choline acetyltransferase (ChAT), with a large multipolar cell body of 23–50 μm in diameter and widespread aspiny dendrites that arborize up to 1 mm (Kimura *et* *al.,* 1981; Bolam *et* *al.,* 1984b; Wilson *et* *al.,* 1990) with 3–6 primary dendrites that extend in a radial pattern (Doig *et* *al.,* 2014). Electron microscopy of rat striatal tissue performed by Doig *et* *al.,* 2010, 2014 indicates that ChIs receive a prominent inhibitory input and that most of excitatory input is from thalamic afferents; a single ChI receives 8450 ± 694 connections of which the majority are symmetric. Moreover, there are approximately three times more vesicular glutamate transporter type 2 (vGLUT2)‐positive thalamic terminals than vesicular glutamate transporter type 1 (vGLUT1)‐positive cortical terminals in an individual ChI (Doig *et* *al.,* 2014). It is important to mention that boutons expressing vGLUT1 and vGLUT2 are the highest in the dorsal one‐third in the rat striatum (Wouterlood *et* *al.,* 2012). However, since vGLUT2 is also expressed in some dopamine terminals in ventral striatum (Stuber *et* *al.,* 2010), it is harder to isolate thalamic inputs.

In spite of the comparative small number of ChIs (Lehmann *et* *al.,* 1979; Bolam *et* *al.,* 1984a; Bennett & Wilson, 1999; Bennett *et* *al.,* 2000; Kreitzer, 2009; Girasole & Nelson, 2015), their long and many branched axons allow a widespread release of ACh (Bolam *et* *al.,* 1984a; Contant *et* *al.,* 1996; Calabresi *et* *al.,* 2000). Initially, ChIs were described as homogeneously dispersed; however, in mice, a greater concentration of ChIs in the dorsomedial compared to ventrolateral areas was observed following a stereological reconstruction (Matamales *et* *al.,* 2016). A correlation between this distribution and the presence of vGLUT1 and vGLUT2 contribute to a possible segregation of function.

Similar to dopaminergic axon varicosities, cholinergic ones, form few structurally defined synaptic connections, therefore favoring a slow cholinergic volume transmission (Descarries *et* *al.,* 1997; Zhou *et* *al.,* 2001; Aznavour *et* *al.,* 2003; Coppola *et* *al.,* 2016; Ovsepian *et* *al.,* 2016; Dunant & Gisiger, 2017). The integration of a striatal cholinergic tone established by volume and synaptic transmission is considered to act within neuronal networks to change their balance of activity to possibly initiate neuronal ensembles with specific functions (Fuxe *et* *al.,* 2012).

### Electrophysiological

The spontaneously active firing characteristic of ChIs ensures the basal cholinergic tone (Kawaguchi *et* *al.,* 1995; Lee *et* *al.,* 1998; Wilson, 2005). These neurons have high input resistance, a broad action potential duration (Wilson *et* *al.,* 1990; Tubert *et* *al.,* 2016), a depolarized, and often changing, resting membrane potential that is usually fixed at −60 mV with a low holding current (Threlfell *et* *al.,* 2012). These interneurons also called ‘tonically active neurons or TANs’ and ‘autonomous pacemakers’ are able to produce action potentials at 2–10 Hz in the absence of synaptic input (Bolam *et* *al.,* 1984a; Wilson *et* *al.,* 1990). Behind this tonic or pacemaking mechanism, it is an interplay of several ionic conductances (Wilson *et* *al.,* 1990; Pisani *et* *al.,* 2007). Their pacemaker cycle begins with an initial tetrodotoxin‐sensitive sodium current‐induced depolarization that leads to calcium influx from Ca_V_2 channels. This first calcium influx in turn activates the calcium and voltage‐activated big potassium currents (BK). This potassium influx contributes to membrane repolarization and the activation of the Ca_V_2.2 current that, in turn, activates the small‐conductance calcium‐activated potassium current (SK). This second potassium current induces a medium duration after‐hyperpolarization (mAHP) of 100–200 ms that defines the spike pattern and spike width (Kawaguchi, 1992; Bennett *et* *al.,* 2000; Goldberg & Wilson, 2005). A decrease in intracellular calcium levels reduces the SK current and consequently the mAHP. The *I*_h_ inward cyclic nucleotide‐gated cation current (HCN) repolarizes the membrane to about −60 mV, with a resulting inactivation of the outward potassium A‐type K_V_4 current. At the end of the cycle, depolarization is slowed down, the persistent sodium current is activated, and the threshold for an action potential is reached, beginning a new sequence (Bennett *et* *al.,* 2000; Goldberg & Wilson, 2005; Deng *et* *al.,* 2007; Pisani *et* *al.,* 2007).

Another feature of ChIs is a long pause in the tonic firing that follows bursts of action potentials. Their intrinsic properties allow ChIs to fire in regular, irregular, and in burst fashion interspersed with long pauses (Bennett *et* *al.,* 2000; Goldberg & Wilson, 2005, 2017; Wilson, 2005; Sanchez *et* *al.,* 2011). During a burst, a subthreshold accumulation of calcium through Ca_V_1 channels recruits an additional potassium current that, in turn, produces a long‐lasting (several seconds) hyperpolarization (sAHP) (Wilson & Goldberg, 2006; Tubert *et* *al.,* 2016).

It is considered that the delta frequency activity of these interneurons results from the combination of synaptic inputs and intrinsic mechanisms (Beatty *et* *al.,* 2015). A muscarinic‐dependent coherence between motor cortex and ChIs can be established following optogenetic stimulation at both beta and low gamma frequencies (Kondabolu *et* *al.,* 2016). The reports on striatal oscillatory activity at different frequencies and the synchronization with other brain regions have been the topic of several recent publications (Brittain & Brown, 2014; Feingold *et* *al.,* 2015; Sharott *et* *al.,* 2017).

Recordings of striatal neurons in behaving primates revealed two cellular striatal populations (Kimura *et* *al.,* 1984): phasic active neurons that show brief action potentials and low spontaneous activity or MSNs (Wilson & Groves, 1981; Apicella, 2017) and TANs that display a broader action potential and tonic spontaneous firing rate (<12 Hz; Kimura *et* *al.,* 1984; Wilson *et* *al.,* 1990; Aosaki *et* *al.,* 1995; Apicella, 2002, 2017; Doig *et* *al.,* 2014). Following electrophysiological criteria, TANs were considered as putative ChIs when antidromic stimulation from globus pallidus (GP) was unable to activate them (Kimura *et* *al.,* 1990, 1996). Moreover, in view of their morphological, electrophysiological, regional, functional, and immunoreactivity similarities, TANs were identified as ChIs (Wilson *et* *al.,* 1990; Aosaki *et* *al.,* 1995; Bennett & Wilson, 1999; Reynolds *et* *al.,* 2004; Inokawa *et* *al.,* 2010; Goldberg & Reynolds, 2011; Bradfield *et* *al.,* 2013; Schulz & Reynolds, 2013; Atallah *et* *al.,* 2014). See Zhang & Cragg (2017) for a review on behavioral studies of TANs and the range of striatal inputs that can modify the pauses.

The fact that the firing properties of TANs are similar to some GABAergic interneurons has created confusion in the proper neuronal differentiation (Berke, 2008; Beatty *et* *al.,* 2012; Gonzales *et* *al.,* 2013; Gonzales & Smith, 2015; Apicella, 2017). It would be best to identify all interneurons, including cholinergic, not only associated with their extracellular electrophysiological characteristics but also with other criteria. The systematic approach to interneuron research being developed (Kepecs & Fishell, 2014; Wamsley & Fishell, 2017) will provide a database of properly classified interneurons (e.g., mRNA‐expression profile). The future will likely bring further determination of their individual electrophysiological characteristics and integrative properties.

## Afferents to cholinergic interneurons

**Figure 1:**
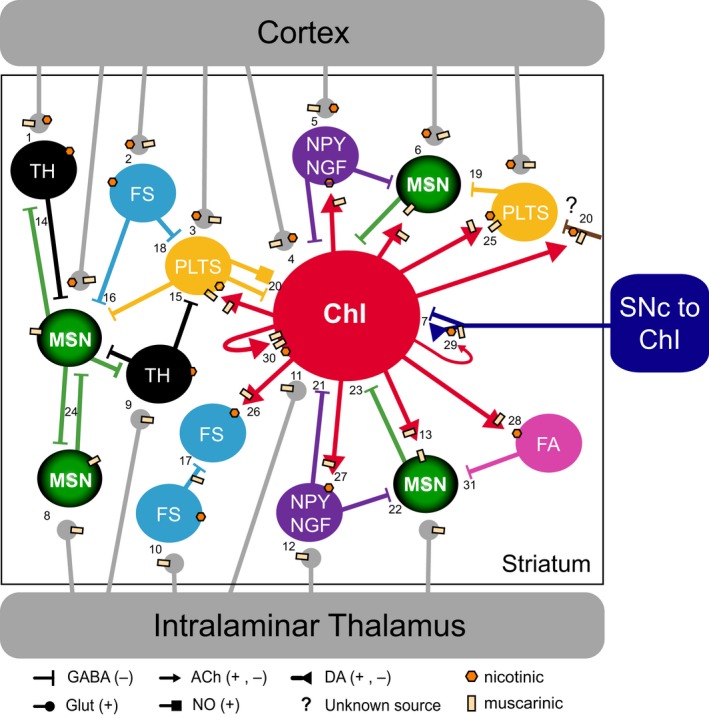
Connectivity of cholinergic interneurons in striatal microcircuits. Afferents from thalamus and cortex initiate direct glutamate‐induced postsynaptic activity in cholinergic and GABAergic interneurons (TH, PLTS, NPY‐NGF, FS subtypes) and in MSNs. ChI connectivity is reciprocal with other ChIs, PLTS, NPY‐NGF interneurons and with MSNs. Unidirectional connections from ChIs are to FA. Intrastriatal unidentified GABAergic terminals are contacted by ChIs expressing nicotinic and muscarinic receptors. These terminals could be dopaminergic (see *Corelease in ChIs*) or GABAergic arkypallidal (*Extrastriatal: GABAergic*). Synaptic connections between ChIs and FS are weak at best and probably FS to ChI connectivity does not exist. Reciprocal connectivity of MSNs with other MSNs and TH interneurons is also illustrated. For simplicity, only the dopaminergic input from SNc to ChIs is illustrated. Abbreviations of interneurons: ChI—cholinergic; PLTS—persistent low‐threshold spiking; NPY‐NGF—neuropeptide‐Y expressing neurogliaform; FA—fast adapting; FS—fast spiking, TH—tyrosine‐hydroxylase. See Table 1 for the numbers associated to connections.

ChIs display symmetric (inhibitory) and asymmetric (excitatory) synaptic specializations, from GABA/substance P and glutamate/dopamine terminals, respectively (Kawaguchi, 1992; Bergson *et* *al.,* 1995; Yan *et* *al.,* 1997; Koos & Tepper, 2002; Zheng & Wilson, 2002; Maurice *et* *al.,* 2004; Lim *et* *al.,* 2014; Munoz‐Manchado *et* *al.,* 2016). Here, we give examples of the established connectivity of ChIs, local neurons, and afferents to striatal microcircuits (Fig. 1; Table 1).

**Table 1:** References supporting connectivity illustrated in Fig. 1

#	From	To	References
1	Cortex	TH	Ibanez‐Sandoval *et* *al*. (2010)
2	Cortex	FS	Bennett & Bolam (1994); Mallet *et* *al*. (2005); Fino *et* *al*. (2008)
3	Cortex	PLTS	Fino *et* *al*. (2009); Ibanez‐Sandoval *et* *al*. (2011)
4	Cortex	ChIs	Lapper & Bolam (1992); Ding *et* *al*. (2010); Doig *et* *al*. (2014); Guo *et* *al*. (2015)
5	Cortex	NPY/NGF	Ibanez‐Sandoval *et* *al*. (2011); Assous *et* *al*. (2017)
6	Cortex	MSN	Somogyi *et* *al*. (1981); Barral *et* *al*. (1999); Ding *et* *al*. (2010); Doig *et* *al*. (2010); Huerta‐Ocampo *et* *al*. (2014)
7	SNc	ChIs	Chuhma *et* *al*. (2014), Straub *et* *al*. (2014)
8	Thalamus	MSN	Ding *et* *al*. (2010); Doig *et* *al*. (2010); Dube *et* *al*. (1988); Sadikot *et* *al*. (1992); Huerta‐Ocampo *et* *al*. (2014)
9	Thalamus	TH	Assous *et* *al*. (2017)
10	Thalamus	FS	Kita (1993)
11	Thalamus	ChIs	Lapper & Bolam (1992); Ding *et* *al*. (2010); Doig *et* *al*. (2010)
12	Thalamus	NPY/NGF	Assous *et* *al*. (2017)
13	ChIs	MSN	Bolam *et* *al*. (1986); Bernard *et* *al*. (1992); Lapper & Bolam (1992); Hersch & Levey (1995); Bennett & Wilson (1998); Alcantara *et* *al*. (2001); Yan *et* *al*. (2001); Chuhma *et* *al*. (2011); Goldberg & Reynolds (2011); Goldberg *et* *al*. (2012); Gonzales *et* *al*. (2013); Guo *et* *al*. (2015); Phelps *et* *al*. (1985); Izzo & Bolam (1988)
14	TH	MSN	Ibanez‐Sandoval *et* *al*. (2010); Freund *et* *al*. (1984)
15	TH	PLTS	Assous *et* *al*. (2017)
16	FS	MSN	Kita (1993); Koos & Tepper (1999); Gittis *et* *al*. (2010); Bennett & Bolam (1994)
17	FS	FS	Koos & Tepper (1999); Gittis *et* *al*. (2010)
18	FS	PLTS	Gittis *et* *al*. (2010); Szydlowski *et* *al*. (2013)
19	PLTS	MSN	Kawaguchi (1993); Gittis *et* *al*. (2010)
20	PLTS	ChIs	Elghaba *et* *al*. (2016); Straub *et* *al*. (2016)
21	NPY/NGF	ChIs	Assous *et* *al*. (2017)
22	NPY/NGF	MSN	English *et* *al*. (2012)
23	MSN	ChIs	Mulder *et* *al*. (1984); Bolam *et* *al*. (1986); Le Moine *et* *al*. (1994); Aosaki & Kawaguchi (1996); Bell *et* *al*. (1998); Pickel *et* *al*. (2000); Jabourian *et* *al*. (2005); Perez *et* *al*. (2007); Govindaiah *et* *al*. (2010); Gonzales *et* *al*. (2013); Ponterio *et* *al*. (2013); Gonzales & Smith (2015)
24	MSN	MSN	Wilson & Groves (1980); Taverna *et* *al*. (2008); Burke *et* *al*. (2017)
25	ChIs	PLTS	Vuillet *et* *al*. (1992); Elghaba *et* *al*. (2016)
26	ChIs	FS	Chang & Kita (1992); Koos & Tepper (2002); English *et* *al*. (2012)
27	ChIs	NPY/NGF	Assous *et* *al*. (2017)
28	ChIs	FA	Faust *et* *al*. (2015); Faust *et* *al*. (2016)
29	ChIs	Dopamine terminals	Jones *et* *al*. (2001); Zoli *et* *al*. (2002); Salminen *et* *al*. (2004); Exley & Cragg (2008); Gotti *et* *al*. (2009); Threlfell *et* *al*. (2012); Gonzales & Smith (2015)
30	ChIs ChIs	Autoreceptors ChIs	Ding *et* *al*. (2006); Pakhotin & Bracci (2007)
31	FA	MSN	Faust *et* *al*. (2015); Faust *et* *al*. (2016)

These selected references by no means reflect all the evidence gathered through more than 40 years of research, apologies for unintended omissions.

### Intrastriatal

A key intrastriatal microcircuit is formed by connections between MSNs, interneurons, and ChIs. In general, 60% of the total intrastriatal synaptic contacts are GABAergic and somatodendritic (Gonzales *et* *al.,* 2013; Gonzales & Smith, 2015). Medium size spiny neurons that release substance P and dynorphin (Bolam *et* *al.,* 1986; Pickel *et* *al.,* 2000; Perez *et* *al.,* 2007) or enkephalin (Le Moine *et* *al.,* 1994; Jabourian *et* *al.,* 2005) contact and modulate ChIs. Importantly, opposite actions are described for their effects: excitatory for substance P (Aosaki & Kawaguchi, 1996; Bell *et* *al.,* 1998; Perez *et* *al.,* 2007; Govindaiah *et* *al.,* 2010) and a powerfully inhibitory for opioid agonists (Mulder *et* *al.,* 1984; Jabourian *et* *al.,* 2005; Ponterio *et* *al.,* 2013). Axon collaterals of MSNs contact ChIs (Bolam *et* *al.,* 1986; Lapper & Bolam, 1992; Bennett & Wilson, 1998; Gonzales *et* *al.,* 2013; Guo *et* *al.,* 2015). In rhesus monkeys, striatal output neurons of both types contact ChIs (Gonzales *et* *al.,* 2013); however, in rodents, substance P containing terminals of dMSNs contact ChIs (Bolam *et* *al.,* 1986; Martone *et* *al.,* 1992). Microcircuits where ChIs are connected among themselves through GABAergic interneurons can be seen when a single action potential produced in a ChI evokes nAChR‐mediated polysynaptic GABA_A_ inhibitory postsynaptic currents (Sullivan *et* *al.,* 2008). Connectivity with an incidence of 9 ChIs to 12 MSN has been observed following MSN optogenetic stimulation (Chuhma *et* *al.,* 2011). Some interactions of ChIs occur between reciprocally connected ChIs (Pakhotin & Bracci, 2007) and with the GABAergic NPY‐low threshold spiking subtype (Vuillet *et* *al.,* 1992). It would be important to determine if striatal GABA_A_ receptors contain the δ subunit that has been shown to be persistently active and to control presynaptic excitability in the spinal cord (Liu *et* *al.,* 2017).

### Extrastriatal

## GABAergic

**Figure 2:**
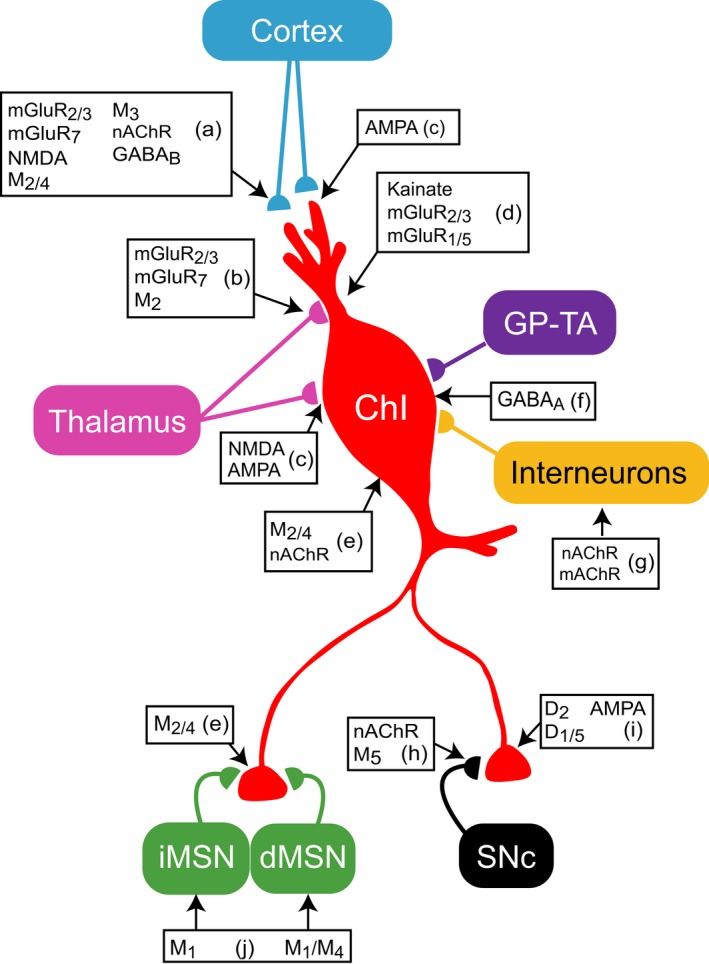
Influence of afferents on cholinergic activity and release. As mentioned in the text, pre‐ and postsynaptic auto‐ and heteroreceptors to ChIs and their afferents can selectively affect the spatial and temporal release of ACh with important functional consequences. The participation of different types of glutamate receptors not only modulates ChI activity and ACh release but also exerts a fine control over dopamine release and other interneuronal and MSN activity. Coincident afferent striatal activation can induce short‐ and long‐term changes in ACh release important in the expression of striatal functions; in this way, ChIs, although few in number, are centrally positioned to likely control neuronal activity using wired and volume transmission. See Table 2 for the letters associated to the references of postsynaptic and presynaptic auto‐ and heteroreceptors.

Extrastriatal GABAergic afferents arrive to striatum from three different GABAergic afferents, two from GP and one from substantia nigra par compacta (SNc) (Fig. 2; Table 2). In GP, the arkypallidal‐type A (GP‐TA) and the prototypic‐type I (GP‐TI) have been classified by electrophysiological (Mallet *et* *al.,* 2008), anatomical (Bevan *et* *al.,* 1998), and molecular (Mallet *et* *al.,* 2012; Mastro *et* *al.,* 2014; Abdi *et* *al.,* 2015) techniques. The GP‐TA express preproenkephalin gene and FoxP2 or Meis2 transcription factors (Abdi *et* *al.,* 2015) and contact cholinergic, nitric oxide synthase (NOS) interneurons, and MSNs (Mallet *et* *al.,* 2012). SNc terminals that corelease dopamine and GABA synaptically modify the activity of ChIs (Chuhma *et* *al.,* 2014; Straub *et* *al.,* 2014), both types of MSNs, and other interneurons (Tritsch & Sabatini, 2012).

**Table 2:** References supporting connectivity illustrated in Fig. 2

Letter	References
a	Hersch *et* *al*. (1994); Testa *et* *al*. (1994); Calabresi *et* *al*. (1998c); Hernandez‐Echeagaray *et* *al*. (1998); Barral *et* *al*. (1999); Bell *et* *al*. (2002); Pisani *et* *al*. (2002); Conn *et* *al*. (2005); Ribeiro (2005); Pakhotin & Bracci (2007); Martella *et* *al*. (2009); Campos *et* *al*. (2010); Ding *et* *al*. (2010); Atwood *et* *al*. (2014); Pancani *et* *al*. (2014); Kupferschmidt & Lovinger (2015); Shen *et* *al*. (2015); Banerjee *et* *al*. (2016); Howe *et* *al*. (2016)
b	Testa *et* *al*. (1994); Bell *et* *al*. (2002); Martella *et* *al*. (2009); Johnson *et* *al*. (2017); Pisani *et* *al*. (2002); Conn *et* *al*. (2005); Ding *et* *al*. (2010); Atwood *et* *al*. (2014); Ribeiro *et* *al*. (2017)
c	Di Chiara *et* *al*. (1994); Consolo *et* *al*. (1996); Calabresi *et* *al*. (1998b); Vorobjev *et* *al*. (2000); Cepeda *et* *al*. (2001); Deng *et* *al*. (2010); Kosillo *et* *al*. (2016)
d	Calabresi *et* *al*. (1998a); Calabresi *et* *al*. (1999a) Bell *et* *al*. (2002); Conn *et* *al*. (2005); Mitrano & Smith (2007); Ribeiro *et* *al*. (2017)
e	Hersch *et* *al*. (1994); Yan & Surmeier (1996); Bernard *et* *al*. (1998); Azam *et* *al*. (2003); Ding *et* *al*. (2006); Eskow Jaunarajs *et* *al*. (2015)
f	Yan *et* *al*. (1997); Bennett & Wilson (1998)
g	Bernard *et* *al*. (1998); Sullivan *et* *al*. (2008); English *et* *al*. (2012); Eskow Jaunarajs *et* *al*. (2015); Elghaba *et* *al*. (2016); Straub *et* *al*. (2016); Assous *et* *al*. (2017)
h	Weiner *et* *al*. (1990); Jones *et* *al*. (2001); Zhou *et* *al*. (2001); Zoli *et* *al*. (2002); Salminen *et* *al*. (2004); Gotti *et* *al*. (2009); Livingstone & Wonnacott (2009); Chuhma *et* *al*. (2014); Foster *et* *al*. (2014); Straub *et* *al*. (2014); Wang *et* *al*. (2014); Gonzales & Smith (2015); Howe *et* *al*. (2016); Garcao *et* *al*. (2014)
i	Richfield *et* *al*. (1989); Bergson *et* *al*. (1995); Yan *et* *al*. (1997); Yan & Surmeier (1997); Aosaki *et* *al*. (1998); Alcantara *et* *al*. (2003); Centonze *et* *al*. (2003a); Cabrera‐Vera *et* *al*. (2004); Maurice *et* *al*. (2004); Ding *et* *al*. (2006); Deng *et* *al*. (2007); Ding *et* *al*. (2010); Ding *et* *al*. (2011)
j	Bernard *et* *al*. (1992); Hersch *et* *al*. (1994); Santiago & Potter (2001); Yan *et* *al*. (2001); Perez‐Rosello *et* *al*. (2005); Hernandez‐Flores *et* *al*. (2015)

These selected references by no means reflect all the evidence gathered through more than 40 years of research, apologies for unintended omissions.

## Glutamatergic

Presynaptic regulation of ACh release has an important function in control of the excitability in striatal microcircuits (Fig. 2). The regulation of dopamine release mediated by a glutamate‐ACh link has become important, and metabotropic glutamate (mGlu) receptors are being explored as potential targets for the treatment of neurodegenerative diseases (Ribeiro, 2005). As indicated before, glutamatergic fibers from both cortex and intralaminar thalamus form asymmetric synaptic contacts on striatal ChIs but with a higher proportion of synaptic contacts from thalamic inputs (Doig *et* *al.,* 2014). Cortical axons contact distal striatal dendrites, and thalamic axons contact striatal somas and dendritic shafts (Lapper & Bolam, 1992). In primates, approximately 20% of synaptic connections to ChIs are presumed glutamatergic and localized on the distal dendrites (Gonzales *et* *al.,* 2013; Gonzales & Smith, 2015), and in rodents, the soma and proximal dendrites of ChIs are the targets of glutamatergic input (Doig *et* *al.,* 2014). However, both cortical and thalamic stimulation induces short latency responses in ChIs and effects of the different afferent synaptic locations have been explored. Compared to responses induced by thalamic stimulation, cortical responses are less robust and attenuate if the stimulation is repeated (Doig *et* *al.,* 2014). These differences could mediate the length of the pause and strength of the rebound; sustained thalamic input seems to keep cholinergic firing followed by long pauses with no rebound. Moreover, the variable intrinsic activity of ChIs seems more important than the location of the afferents in the moment‐to‐moment variability in the size of neuronal recruitment (Kosillo *et* *al.,* 2016). The section *‘*Influence of cholinergic interneurons within the striatal microcircuits: dopaminergic terminals’ describes other experiments that have contributed to clarify the role of glutamate receptors selectively activated by cortical or thalamic afferents.

ChIs express postsynaptic and presynaptic ionotropic and metabotropic glutamate heteroreceptors (Testa *et* *al.,* 1994; Landwehrmeyer *et* *al.,* 1995; Bell *et* *al.,* 2002; Deng *et* *al.,* 2010). A membrane depolarization (Vorobjev *et* *al.,* 2000; Cepeda *et* *al.,* 2001) and modulatory actions mediated by PKC are observed in ChIs (Di Chiara *et* *al.,* 1994; Calabresi *et* *al.,* 1998a) following the activation of postsynaptic glutamate ionotropic receptors, that is, n‐methyl‐D‐aspartate (NMDA), α‐amino‐3‐hydroxy‐5‐methyl‐4‐isoxazolepropionic acid (AMPA), or kainic acid.

The presynaptic activation of these receptors on ChIs increases ACh release (Consolo *et* *al.,* 1996). In striatal microcircuits, mGluRs modulate excitability and neurotransmitter release (Conn *et* *al.,* 2005). The group‐III member, mGlu_7_, is not expressed in ChIs (Pisani *et* *al.,* 2002) but expressed presynaptically as autoreceptors where it decreases the probability of release and in turn postsynaptic cholinergic excitability (Bell *et* *al.,* 2002). Group‐II mGlu_2/3_ receptors are expressed pre‐ and postsynaptically in ChIs (Testa *et* *al.,* 1994; Bell *et* *al.,* 2002). As presynaptic heteroreceptors, they decrease glutamate release with a consequent depression of excitatory postsynaptic potentials (Martella *et* *al.,* 2009). The mGlu_2/3_ autoreceptors (and GABA_B_ receptors) dampen glutamate release, decrease postsynaptic excitatory responses, and can produce a transient depression (Martella *et* *al.,* 2009) and long‐term depression (LTD) (Kupferschmidt & Lovinger, 2015). Moreover, mGlu_2/3_ receptors are predominantly coupled to G_i/o_ proteins that mediate inhibition of AC activity and also to other cell signaling pathways involved in neuroprotection. For example, extracellular signal‐regulated kinase activation attenuates rotenone toxicity on dopaminergic neurons (Ribeiro, 2005). ChIs also express group‐I mGlu_1/5_ (Bell *et* *al.,* 2002), especially in dendrites (Mitrano & Smith, 2007). The activation of mGlu_1/5_ receptors induces membrane depolarization (Calabresi *et* *al.,* 1999b; Bell *et* *al.,* 2002; Martella *et* *al.,* 2009).

## Dopaminergic

Dopaminergic SNc afferents exert a robust striatal influence due to their tonic spontaneous activity (1‐8 Hz) and broad terminal field arborization (Prensa & Parent, 2001; Schultz, 2007; Matsuda *et* *al.,* 2009); a single dopamine neuron has a dense terminal field that occupies 3% of striatal volume with axonal varicosities forming synapses every 2 μm (Arbuthnott & Wickens, 2007). D_2_ receptors located postsynaptically on ChIs reduce autonomous firing through voltage‐sensitive sodium channels (Maurice *et* *al.,* 2004; Ding *et* *al.,* 2010) or hyperpolarization‐activated HCN currents (Deng *et* *al.,* 2007).

The dopamine–ACh interaction is mediated by D_2_ and D_1/5_ receptors. D_1_/D_5_ subtypes are expressed in dendrites (Bergson *et* *al.,* 1995; Yan & Surmeier, 1997; Yan *et* *al.,* 1997) and D_2_ receptors are located in soma, dendrites, and axons (Alcantara *et* *al.,* 2003). The activation of D_1_/D_5_ receptors in slice preparations enhances ChIs excitability (Centonze *et* *al.,* 2003b; Ding *et* *al.,* 2011). Apparently, a cAMP‐dependent mechanism allows the closure of potassium channels and promotes the opening of nonselective cation channels (Aosaki *et* *al.,* 1998). Cholinergic receptors expressed in the dopaminergic axon terminal fields modulate dopamine release; nAChRs increase dopamine release (Imperato *et* *al.,* 1986; Calabresi *et* *al.,* 1989) whereas presynaptic M_5_ mAChRs reduce it (Foster *et* *al.,* 2014). At the somatodedritic level, both nAChRs and M_5_ mAChR increase spontaneous activity (Foster *et* *al.,* 2014). Other effects on dopamine release mediated by other mAChR subtypes appear related to the stimulation of receptors located in non‐dopaminergic neurons (Zhang *et* *al.,* 2002a).

Using optogenetic stimulation of dopaminergic terminals *in vitro*, a biphasic modulatory action on ChIs was similar to the pause‐rebound response of putative ChIs recorded *in vivo*. This consisted in a decrease in spike rate and a delayed excitatory response that peaked 0.4–0.6 s after stimulation (Straub *et* *al.,* 2014).

Although presynaptic D_2_ receptors on ChIs limit ACh release through voltage‐gated Ca_V_2 channels, an important control of downstream processes is also provided by the regulators of G‐protein signals (RGS) (Anderson *et* *al.,* 2009). Ding *et* *al*. (2006) observed that following dopamine depletion, M_4_ rather than D_2_ receptors alter signaling in ChI. In the absence of dopamine, M_4_ autoreceptors suffer the attenuation of Ca_v_2 channel opening and pacemaking by upregulation of the expression of RGS9. Consistently, significant decreases of RGS9 protein concentration and mRNA were observed in dopamine depleted animals following L‐DOPA treatment (Yin *et* *al.,* 2011).

## Other afferents

Axon terminals releasing serotonin, histamine, or adenosine are known to modulate the activity of ChIs. Serotonin afferents from the dorsal raphe nucleus (Miguelez *et* *al.,* 2014) induce a direct excitatory effect on ChIs through 5‐HT_2_ (Blomeley & Bracci, 2005) and 5‐HT_6_ receptors (Bonsi *et* *al.,* 2007). Similarly, histamine‐containing afferents from the hypothalamic tuberomamillary nucleus (Bolam & Ellender, 2016) depolarize ChIs by the activation of GPCR histamine receptor type 1 (H_1_) (Bell *et* *al.,* 2000). In nucleus accumbens, the activation of ChI H_3_ receptors decreases their spontaneous activity, but this effect can only be observed in accumbens since striatum does not seem to express this histamine receptor subtype (Varaschin *et* *al.,* 2018). The purine nucleoside, adenosine, is released by neurons and glia. Of the four subtypes of GPCR adenosine receptors in brain, the A_2A_ subtype is mostly expressed in striatum (Dunwiddie & Masino, 2001). Striatal A_1_ and A_2A_ receptors in ChI are potent regulators of striatal ACh release with opposite effects (Preston *et* *al.,* 2000; Song *et* *al.,* 2000). Concomitant dopamine D_2_ and A_2A_ receptor stimulation inhibits ACh release (Song & Haber, 2000; Tozzi *et* *al.,* 2011). Moreover, adenosine reverses N‐type calcium currents in ChIs and both MSNs through membrane G‐protein pathways (Song *et* *al.,* 2000; Hernandez‐Gonzalez *et* *al.,* 2014).

## Influence of cholinergic interneurons within striatal microcircuits

**Figure 3:**
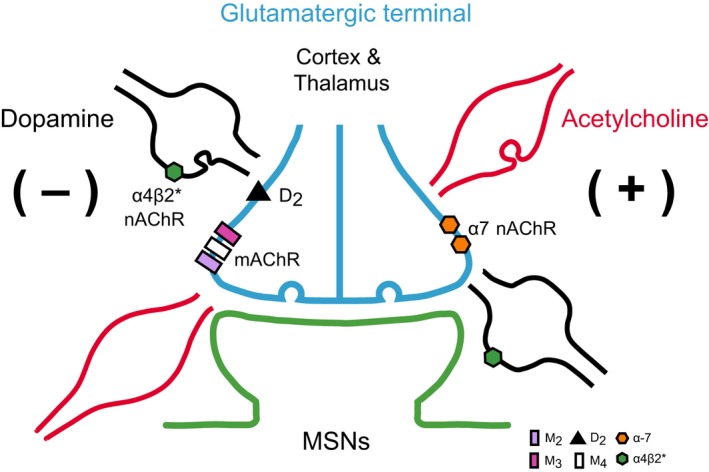
Presynaptic muscarinic and nicotinic control of striatal glutamate release. Illustrated are the effects of ACh release within striatal microcircuits as discussed in the sections ‘dopaminergic terminals’ and ‘glutamatergic terminals’. The cartoon depicts Right: An increase in glutamate release mediated by presynaptic α7 nAChR on glutamate terminals. Left: A decrease in glutamate release mediated by two mechanisms: (i) a direct effect of ACh on presynaptic mAChRs (M_2_, M_3_, and M_4_), or (ii) an indirect effect of ACh mediated by an increase in dopamine due to activation of α4β2* nAChRs on dopamine terminals. Dopamine action on inhibitory D_2_ receptors on glutamate terminals reduces glutamate release. Such a complex action on the same terminal as depicted in (Fig. 2) [if indeed the receptors are coexpressed on single terminals] suggests either that fine control of the concentration of glutamate or the precise timing of it is important for MSN activity. The second, more indirect, inhibition by α4β2* nAChRs on dopamine terminals may be an important source of the increased activity in striatum in the absence of dopamine when such inhibition would be removed. The symbol code depicts the receptor types and their location.

In spite of their relative small number, ChIs within the striatal microcircuits form enmeshed axonal projections with an extensive neuromodulatory presynaptic and postsynaptic effect (Descarries *et* *al.,* 1997; Descarries & Mechawar, 2000) and most likely, interact with all neuronal elements through synaptic and volume transmission (Threlfell & Cragg, 2011). The modulation of striatal microcircuits by ChIs is exemplified in studies involving neuronal excitability and neurotransmitter release (Figs 2 and 3).

## Medium spiny neurons

ChIs synapse onto dendritic spines (Hersch & Levey, 1995; Alcantara *et* *al.,* 2001) of iMSN and dMSNs (Izzo & Bolam, 1988; Bernard *et* *al.,* 1992; Yan *et* *al.,* 2001; Goldberg *et* *al.,* 2012). In electrophysiologically identified MSNs, ACh evokes complex excitatory actions by direct modulation of several ionic currents, mainly potassium, sodium, and calcium (Pineda *et* *al.,* 1995; Perez‐Rosello *et* *al.,* 2005; Shen *et* *al.,* 2007; Carrillo‐Reid *et* *al.,* 2009). Both dMSNs and iMSNs express M_1_ receptors, and their activation increases neuronal excitability by the enhancement of the persistent sodium conductance and by directly or indirectly depressing potassium currents (Akins *et* *al.,* 1990; Galarraga *et* *al.,* 1999; Figueroa *et* *al.,* 2002; Perez‐Rosello *et* *al.,* 2005; Shen *et* *al.,* 2005, 2007; Carrillo‐Reid *et* *al.,* 2009; Goldberg *et* *al.,* 2012; Perez‐Ramirez *et* *al.,* 2015). Both M_1_, M_4_ receptors are expressed in dMSNs (Santiago & Potter, 2001; Yan *et* *al.,* 2001; Goldberg *et* *al.,* 2012), and the activation of M_4_ with muscarine increases MSN excitability by enhancing Ca_V_1 channels (Hernandez‐Flores *et* *al.,* 2015).

A strong depolarization induced by glutamatergic striatal afferents triggers a postsynaptic release of endocannabinoids (eCB). CB_1_ receptors are one of the most abundant GPCRs in the central nervous system and are located at excitatory and inhibitory presynaptic and axonal compartments. CB_2_ receptors are primarily localized in microglia (Kendall & Yudowski, 2016). CB_1_ receptors are coupled to pertussis toxin‐sensitive G_i/o_ type G‐proteins, and their striatal activation results in a presynaptic long‐term depression in corticostriatal synapses (Adermark & Lovinger, 2007).

ChIs are also important regulators of striatal eCB. ACh produces an indirect modulatory effect in the regulation of striatal plasticity through the eCB system (Oldenburg & Ding, 2011). At inhibitory synapses, M_1_ receptor stimulation promotes eCB production and retrograde activation of CB_1_R that suppresses the inhibitory synaptic transmission. In contrast, at excitatory glutamatergic synapses, an M_1_ agonist reduces postsynaptic Ca_v_1.3 currents that, in turn, decrease eCB production and activation of presynaptic CB_1_R (Wang *et* *al.,* 2006; Narushima *et* *al.,* 2007). Low to moderate activation of corticostriatal afferents *in vitro* (5 Hz/60 s) produces a long‐lasting disinhibition of synaptic input that complies with all the requisites for the induction of striatal high‐frequency stimulation‐induced LTD (Calabresi *et* *al.,* 1992; Adermark & Lovinger, 2007; Kreitzer & Malenka, 2008).

The *in vitro* long‐lasting disinhibition of synaptic input induced by corticostriatal afferents can be prevented with an antagonist to the non‐α7 nAChR; moreover, a nicotine‐induced facilitation of eCB‐LTD is occluded by the dopamine receptor agonist quinpirole and by the mAChR antagonist scopolamine (Adermark *et* *al.,* 2018). Using a slightly different paradigm to induce LTD in MSNs (i.e., direct activation of mGlu_1_ with the agonist (S)‐3,5‐Dihydroxy‐phenylglycine (50 μm) plus postsynaptic depolarization to −50 mV), selective optogenetic stimulation of cortical or thalamic afferents revealed that cortical, but not thalamic afferent stimulation, induces a significant eCB‐LTD accompanied by a decreased probability of presynaptic release. Double immunohistochemistry of CB_1_R and vGLUT1 or vGLUT2 indicates cortical vGLUT1 terminals colocalize ≈4 times more with CB_1_ (Wu *et* *al.,* 2015).

Long‐term changes in striatal excitability by cortical and thalamic axonal stimulation could be related to their different proposed functions: goal‐directed behavior for cortical afferents (Graybiel, 1995) and attention and arousal for thalamic afferents (Alloway *et* *al.,* 2017).

The interrelation between MSNs, glutamatergic cortical afferents, ChIs, and presynaptic action on dopamine terminals opens deliberation as to whether other receptors located in these microcircuits have a direct or indirect effect on MSNs (see Fig. 3).

## GABAergic interneurons

Symmetrical synapses between labeled MSNs and interneurons are observed in striatum (Bennett & Bolam, 1994). GABAergic interneurons may not only be influenced by cortical or thalamic inputs but also by local ChIs. For example, excitatory activation of GABAergic interneurons by nAChR are frequently reported (Sullivan *et* *al.,* 2008; English *et* *al.,* 2012; Luo *et* *al.,* 2013; Ibanez‐Sandoval *et* *al.,* 2015; Munoz‐Manchado *et* *al.,* 2016).

Within striatal microcircuits, there is a neuronal chain that follows glutamatergic input to ChIs, then inputs to NPY‐NGF interneurons, and finally GABAergic input to MSNs evidenced *in vitro* following multicellular recordings and calcium imaging. The activation of nAChRs on GABAergic interneurons induces a global decrease in neuronal activity indicating a general activation of inhibitory GABAergic interneurons (Plata *et* *al.,* 2013). Similarly following synchronized activation of ChIs, the GABAergic NPY‐NGF subtype produces the inhibition of MSNs mediated by ChI to GABAeric interneuronal synapses and then to MSN (Faust *et* *al.,* 2015, 2016). The recurrent inhibition of ChIs is sensitive to nicotinic antagonists therefore not mediated by the GABAergic interneuron (Sullivan *et* *al.,* 2008; English *et* *al.,* 2012). Optogenetic activation of glutamatergic thalamic afferents to ChIs provides a nicotinic excitatory input to NPY‐NGF interneurons that in turn modulate MSN activity (Assous *et* *al.,* 2017). Within this neuronal chain, it is still unknown if other interneurons such as the FA subtype also participate.

Additionally, persistent low‐threshold spiking (PLTS) interneurons are highly excited by cortical afferents (Assous *et* *al.,* 2017) and are directly and indirectly modulated by both nACh and mACh receptors. The amplitude of striatal intracellular responses mediated by GABA decreases in the presence of muscarine and ACh (Sugita *et* *al.,* 1991).

A mutual excitatory interaction exits between ChIs and PLTS: ChIs acting on nAChR directly excite PLTS interneurons and indirectly through mAChR on unidentified GABAergic terminals. The net effect of a tonic cholinergic action on the GABAergic interneurons is inhibitory as both nicotinic and muscarinic antagonists reverse the inhibition (Elghaba *et* *al.,* 2016). This evidence suggests that interconnected ChIs and GABAergic interneurons form a subcircuit that could allow flow of information independent of classical inputs such as MSNs to FSI (Luo *et* *al.,* 2013; Faust *et* *al.,* 2015, 2016).

## Dopaminergic terminals

It is clear that synaptic release modulated at the terminal level, independent of the cell body, is a major component of the striatal microcircuits (Rice & Cragg, 2008). It has been calculated that within a sphere of striatal tissue of 20 μm in diameter, point‐to‐point synaptic communication for dopamine and ACh terminals takes place. Axons of dopamine and cholinergic neurons contribute each ≈ 400 terminals that are intermingled with other 2000–4000 unidentified terminals (Descarries *et* *al.,* 1997). Such observations led Agnati *et* *al*. (1986), as quoted by Fuxe *et* *al*. (2013), to propose the concept of volume transmission as a non‐junctional mode of intercellular communication. By modeling striatal dopamine spillover after quantal release, Rice & Cragg (2008) concluded that uptake does not limit the initial overflow from an extrasynaptic or synaptic release site, resulting in the formation of a cloud of dopamine that can reach extrasynaptic dopamine receptors which are more abundant than the synaptic receptors.

Studies of cholinergic modulation of dopaminergic terminals suggest that ACh diminishes dopamine release via nAChRs located on dopamine terminals (Rice *et* *al.,* 2011); however, when dopamine release and the activity of ChIs could be simultaneously monitored with fast scan voltammetry, a synchronous activation of ChIs increased striatal dopamine release; for references, see Cachope & Cheer (2014). Therefore, endogenous release of ACh directly triggers striatal dopamine release (Cachope *et* *al.,* 2012) and ChIs synchronized by their thalamic input promote dopamine release (Threlfell *et* *al.,* 2012). The prolonged debate about the interrelation between dopamine and ACh release has been slowly resolving, as more data are gathered. We now know that presynaptic nAChRs are highly expressed on striatal dopaminergic terminals (Jones *et* *al.,* 2001; Zhou *et* *al.,* 2001; Zoli *et* *al.,* 2002; Salminen *et* *al.,* 2004; Gotti *et* *al.,* 2009; Livingstone & Wonnacott, 2009; Garcao *et* *al.,* 2014; Wang *et* *al.,* 2014; Gonzales & Smith, 2015; Howe *et* *al.,* 2016), and that their activation facilitates dopamine release (Exley & Cragg, 2008).

Combined light activation of dopamine terminals and chemogenetic stimulation of ChI potentiates dopamine release (Aldrin‐Kirk *et* *al.,* 2018). Moreover, a neurotoxic dopamine depletion plus chemogenetic activation of ChIs *in vivo* increases the use of previously akinetic forelimbs induced by a low dose of L‐DOPA; however, the activation of ChI combined with a D_2_ agonist (quinpirole), but not a D_1_ agonist, increases the L‐DOPA‐induced abnormal involuntary movements (Aldrin‐Kirk *et* *al.,* 2018). This is congruent with other observations of exacerbation of dyskinesias by D_2_ agonists in mice (Alcacer *et* *al.,* 2017) and increases in dyskinesias seen by the activation of M_1_ receptors on dMSN in combination with presynaptic M_2_ blockade (Bernard *et* *al.,* 1992; Yan *et* *al.,* 2001).

When considering microcircuits, different affinities or the complete absence of ACh (in knockout mice) can produce different modulatory effects. For example, a low affinity α7‐containing nAChR will quickly become desensitized with a resulting decrease in cholinergic modulation; on the contrary, a high affinity α4β2*‐containing nAChR will desensitize more slowly, with a resulting increase in modulatory effect of ACh. Moreover, a ChAT knockout results in mice with no ChIs and produces increased phasic‐to‐tonic dopamine signal with altered dopaminergic and glutamatergic tone (Patel *et* *al.,* 2012).

The participation of corticostriatal and thalamostriatal afferents on dopamine release has been clarified using selective optogenetic activation; increases in dopamine release by the corticostriatal terminal field are mediated by nAChR but modulated by mAChR. Moreover, the increase in dopamine release results from the action of AMPA receptors on ChIs that promote short‐latency action potentials. Dopamine release driven by thalamostriatal afferents involves additional activation of NMDA receptors and action potential generation over longer timescales (Kosillo *et* *al.,* 2016).

If the presence of NMDA receptors in thalamic afferents is observed, it would be interesting to know if they act as ‘sniffers’ of spillover glutamate release, have neurotrophic/neuroprotective function, or are involved in the modulation of postsynaptic responses.

## Glutamatergic terminals

As mentioned before, striatal glutamatergic afferents arrive from cortex and thalamus (Ding *et* *al.,* 2010; Doig *et* *al.,* 2014), and presynaptic mAChRs (subtypes M_1_, M_2_, M_3_, M_4_) are located on axon terminals (Hersch *et* *al.,* 1994). Electrophysiological *in vitro* recordings of striatal slices have been useful to clarify their inhibitory role in the modulation of presynaptic release from excitatory terminals to MSNs and their participation in striatal microcircuits.

Stimulated release of glutamate reduces responses to field pair‐pulse stimulation (Barral *et* *al.,* 1999) and random synaptic events (Hernandez‐Echeagaray *et* *al.,* 1998). Furthermore, pair recordings of interactions between ChIs and MSNs indicate that spontaneous activity of ChIs decreases the amplitude of the MSN intracellularly induced EPSC and that M_2_/M_4_ antagonists prevents the decrease (Pakhotin & Bracci, 2007). Similarly, the activation of M_4_ presynaptic receptors with a positive allosteric modulator decreases glutamate release with a consequent reduction in postsynaptic excitatory currents in both types of MSNs (Pancani *et* *al.,* 2014). Moreover, mAChR induce presynaptic inhibition of striatal glutamatergic terminals through an action on Ca_v_2 channels (Barral *et* *al.,* 1999) with a consequent decrease in glutamate release at both corticostriatal (Hernandez‐Echeagaray *et* *al.,* 1998; Barral *et* *al.,* 1999; Higley *et* *al.,* 2009) and thalamostriatal terminals on dMSN and iMSN (Ding *et* *al.,* 2010), (Fig. 3).

Apart from the muscarinic action, nAChRs play a bidirectional modulation on corticostriatal glutamate release due to the presynaptic location of α7‐containing nicotinic heteroreceptors on corticostriatal afferents and presynaptic α4β2*‐containing nicotinic heteroreceptors on dopamine afferents that in turn contact corticostriatal terminals. The activation of α7‐containing nicotinic heteroreptors on cortical afferents increases glutamate release (Campos *et* *al.,* 2010; Howe *et* *al.,* 2016), whereas the activation of presynaptic α4β2‐containing nicotinic heteroreceptors on dopamine afferents produces a two‐link chain reaction: first, enhanced dopamine release stimulates presynaptic D_2_ heteroreceptors that in turn produce a decrease in glutamate or ‘brake’ effect (Campos *et* *al.,* 2010; Howe *et* *al.,* 2016). Certainly the affinity of nAChRs and mAChR, their location, physiological properties, and activation state of the terminal field have already begun to explain the spectrum of pre‐ and postsynaptic responses to ACh and for that matter to other neurotransmitter receptors.

The variety of auto‐ and heteroreceptors located presynaptically at synaptic and non‐synaptic locations can selectively affect the spatial and temporal control of spontaneous and action potential‐driven neurotransmitter release, depending on the terminal subtype and their intrinsic activity (Banerjee *et* *al.,* 2016; Pittaluga, 2016). After coincident presynaptic activation, short‐ and long‐term changes in neurotransmitter release can also occur (Atwood *et* *al.,* 2014), but most importantly, the controls on release described in this section reflect a precise receptor‐mediated regulation (Fig. 3).

## Co‐release from cholinergic interneurons

Although it goes against the Dale's principle of one neurotransmitter per neuron, the concept of corelease is now more accepted (Hnasko & Edwards, 2012). The presence of the glutamate type 3 vesicular transporter (vGLUT3) in neurons typically indicates the possibility of corelease (Kljakic *et* *al.,* 2017). In striatum, a high expression of the glutamate transporter vGLUT3 is seen in a population of vesicles that express both vGLUT3 and vesicular acetylcholine transporter (vAChT) (Gras *et* *al.,* 2002; Amilhon *et* *al.,* 2010; Kljakic *et* *al.,* 2017). Striatal corelease of ACh and glutamate has been determined following two main strategies: electrophysiological and genetic manipulation. Following the electrophysiological approach, there are two studies: one reports that optical stimulation of ChIs induces in MSNs two glutamate‐dependent responses (Higley *et* *al.,* 2011) and another reports that ACh release following synchronous ChIs triggers an action potential‐independent presynaptic release of GABA colocalized in dopaminergic terminals (Nelson *et* *al.,* 2014). With the genetic approach, it was observed that following the deletion of the vAChT gene and subsequent elimination of ACh release, alterations in gross motor skills and in performance attributed to ACh, are still present most likely as a consequence of coreleased glutamate (Guzman *et* *al.,* 2011).

Several questions must be answered regarding this topic: Does corelease for both neurotransmitters occur at the same time? Is release differentially regulated? Is release spatially coupled? How does the presence of two neurotransmitters contribute to microcircuits function? Does the ratio neurotransmitters change?

## Striosome and matrix compartments

Almost 40 years ago, Graybiel & Ragsdale (1978) reported two distinct densities or compartments in the distribution of AChE in the striatum of primates and cats. These two compartments are called striosomes or patches, and matrix. Striosomes receive dopamine afferents from SNc and glutamatergic afferents from medial prefrontal, anterior cingulate, orbitofrontal, and anterior insular cortices (Benarroch, 2016). Stereological analysis in humans finds a differential distribution of ChIs with most of them located in the periphery of the striosomes (Bernacer *et* *al.,* 2007). Similarly in rodents, ChIs are found in the border of striosomes (Kubota & Kawaguchi, 1993) with extended processes into both compartments (Kubota & Kawaguchi, 1993). In recent reviews, ChIs are described as preferentially located in the matrix (Crittenden & Graybiel, 2011; Crittenden *et* *al.,* 2017). Using new tools, attempts to exclusively stimulate one compartment *in vitro* are clarifying the location of ChIs. Whole‐cell patch recordings of ChIs with *a posteriori* identification of their compartment location revealed that GABAergic currents mediated by nAChRs are more frequently observed in the matrix than the striosome (Inoue *et* *al.,* 2016), and the photoactivation of the matrix compartment with independent local stimulation and patch‐clamp recordings revealed lack of synaptic connectivity between matrix and striosomes (Lopez‐Huerta *et* *al.,* 2016). The presence of ChIs in the areas high in calbindin‐D28K and ChAT (Prensa *et* *al.,* 1999) referred to as the ‘peristriosomal boundary’ reaffirm the location of ChIs between as well as within matrix and striosome compartments (Brimblecombe & Cragg, 2017).

A separation between matrix and striosomes has been established in rats by their different thalamic afferents. Unzai *et* *al*. (2017) reported that striatum and nucleus accumbens receive afferents to the striosome compartment mostly from thalamic midline nuclei, whereas the intralaminar nuclei innervate the matrix compartment. Moreover, whereas most terminal fields form *en passant boutons*, clusters or plexus containing many boutons are observed on terminal fields of the parafascicular nucleus. From the functional point of view, information from these two thalamic areas support the function previously inferred (Vertes *et* *al.,* 2015): limbic (emotional) control for the striosomes and sensorimotor associative for the matrix (White & Hiroi, 1998; Crittenden & Graybiel, 2011; Buot & Yelnik, 2012).

## Participation of cholinergic interneurons in striatal plasticity

It is broadly believed that long‐lasting changes in synaptic efficiency at corticostriatal synapses are the cellular basis of motor learning (Pisani *et* *al.,* 2007; Fino & Venance, 2011; Deffains & Bergman, 2015). These plastic changes have been shown as LTD or as long‐term potentiation (LTP). Early reports of striatal long‐term changes indicated that either LTP or LTD could be produced by high frequency stimulation of cortical or thalamic glutamatergic inputs along with postsynaptic depolarization (Calabresi *et* *al.,* 1992; Lovinger *et* *al.,* 1993; Wickens *et* *al.,* 1996; Centonze *et* *al.,* 2001).

Further studies revealed that the precise timing and order between presynaptic and postsynaptic action potentials dictate the occurrence of either LTP or LTD in the paradigm of spike‐timing‐dependent plasticity (STDP) (Markram *et* *al.,* 2011). As in the case of long‐term changes induced by high‐frequency stimulation, STDP‐induced LTD and LTP was also induced in corticostriatal synapses (Fino *et* *al.,* 2008; Pawlak & Kerr, 2008; Shen *et* *al.,* 2008; Fino & Venance, 2011; Shindou *et* *al.,* 2011; Jedrzejewska‐Szmek *et* *al.,* 2017). Two variables are important for corticostriatal STDP: the frequent *in vivo* bombardment of pre‐ and postsynaptic inputs onto striatal neurons, and the presence of modulators like ACh, dopamine, or serotonin. Extracellular ACh and the level of M_1_ receptor stimulation control the direction of LTP or LTD (Calabresi *et* *al.,* 1999a; Centonze *et* *al.,* 1999). Additionally, cholinergic modulation of eCB synthesis has been linked to these long‐lasting processes (Wang *et* *al.,* 2006; Narushima *et* *al.,* 2007).

The interaction between dopamine and ACh is important in the regulation of MSN excitability and plasticity. It appears that *in vitro* cortical inputs first activate striatal GABAergic FS interneurons, then ChIs, and finally MSNs (Fino *et* *al.,* 2008). This order of events provides a facilitating effect on the MSNs while they receive cortical information and so define the direction of the plasticity (Deffains & Bergman, 2015).

High‐frequency stimulation of cortical or thalamic afferents that synapse onto ChIs leads to an early monosynaptic glutamate‐dependent depolarization (EPSP) followed by an intrastriatal disynaptic GABAergic hyperpolarization (IPSP). In the presence of a GABAergic antagonist, induction of LTP depends on a rise in intracellular calcium and the activation of dopamine D_1_/D_5_ but not D_2_ receptors (Suzuki *et* *al.,* 2001; Bonsi *et* *al.,* 2004; Oswald *et* *al.,* 2015). Moreover, in the absence of a GABAergic antagonist, the LTP of IPSPs recorded in ChIs is presynaptically mediated. The amplitude of each unitary induced IPSP is the same whereas their frequency increases (Suzuki *et* *al.,* 2001; Miura *et* *al.,* 2002). Other experiments suggest that the direction of STDP is determined by the rheobase of the ChIs. If the minimal current amplitude to evoke an action potential is low, LTD is observed in the recorded ChI, whereas LTP is induced if the ChI has a high rheobase (Fino *et* *al.,* 2008; Fino & Venance, 2011).

The study of plasticity of cortical input to striatal GABAergic interneurons is limited due to their low population prevalence and cellular variability. So far, there are a few studies describing STDP on FS or PLTS‐NOS expressing interneurons (Fino *et* *al.,* 2008, 2009). However, with the help of transgenic mice targeting specific interneurons, in the near future, the knowledge in this field will grow.

## ACh and striatal microcircuits

Tonically active ChIs are central in any analysis of the striatal microcircuits and perhaps should be considered within a functional relevant microcircuit. In order to be able to clearly isolate neuronal microcircuits in behaving animals, technical advances are needed. The study of neuronal ensembles was originated by the analysis of the spatiotemporal organization of groups of neurons. To perform the mathematical analyses to reveal interacting neuronal ensembles as multidimensional microcircuits, many neurons should be recorded at once (Yuste, 2015; Carrillo‐Reid *et* *al.,* 2017). Although single cell studies have been valuable revealing direct postsynaptic actions, sometimes conflicting interpretations can occur using the recordings of many interacting cells (Carrillo‐Reid *et* *al.,* 2011). In recent years, these calcium‐imaging techniques have provided the most powerful tool to study spontaneous or drug‐induced neuronal modulation of ≈60–80 striatal neurons for at least 20 min without losing the single cell resolution (Carrillo‐Reid *et* *al.,* 2008).

In the section *‘*Influence of cholinergic interneurons…GABAergic interneurons’, we described that the stimulation of striatal ChIs through nAChRs activation excites GABAergic interneurons that in turn induce recurrent inhibition in themselves and nearby ChIs (Sullivan *et* *al.,* 2008). This effect could conceivably impact the activity in the whole population of striatal neurons. To study this possibility, Plata *et* *al*. (2013) artificially increased activity in the whole population of striatal neurons by bath application of NMDA or a previous chronic dopamine depletion. Under these conditions, it is clear that bath application of 1 μm nicotine clearly inhibits the hyperactive microcircuits.

Excitatory striatal activation of MSNs mediated by mAChRs has also been reported (Lv *et* *al.,* 2017). The activation of M_1_ receptors enhances a persistent sodium current that can synchronize a large population of MSNs (Carrillo‐Reid *et* *al.,* 2009). Moreover, M_1_ receptor activation inhibits the persistent K_V_7‐potassium or the M‐current in the dendritic/spine compartment of MSNs (Perez‐Ramirez *et* *al.,* 2015) and as expected, a specific antagonist of M_1_ receptors also decreases striatal neuronal activity (Hernandez‐Flores *et* *al.,* 2015). The influence of ChI on Kv7 channels is relevant, since these channels are widely expressed and are known to control neuronal excitability, the resting membrane potential, the spiking threshold, and to set the firing frequency within the burst and the subsequent hyperpolarization that follows a burst (Greene & Hoshi, 2017).

## Movement disorders related to cholinergic interneurons

Impairment of striatal ChIs is central in the production of movement disorders (Pisani *et* *al.,* 2007); altered cholinergic signaling is seen in a diverse class of syndromes that include Parkinson's disease (PD; Brichta *et* *al.,* 2013; Kalia *et* *al.,* 2013; Ztaou *et* *al.,* 2016), dystonia (Peterson *et* *al.,* 2010; Eskow Jaunarajs *et* *al.,* 2015; Scarduzio *et* *al.,* 2017), Tourette's syndrome (Xu *et* *al.,* 2015; Albin *et* *al.,* 2017), and Huntington's disease (Di Filippo *et* *al.,* 2007).

Parkinson's disease is a common neurological disorder characterized by a decreased dopamine level. Early clinical and experimental studies revealed that PD was also characterized by increased striatal extracellular levels of ACh (Barbeau, 1962; Cachope & Cheer, 2014). Indeed, the earliest pharmacological treatment of PD consisted of administration of anti‐cholinergic agents (e.g., weak antimuscarinic diphenylhydramine, benztropine, orphenadrine; Fahn, 2014). However, the cumulative effect of anti‐cholinergic medication ‘anti‐cholinergic burden’, and the ‘anti‐cholinergic risk’ associated with a decrease in the use of anti‐cholinergic in old hospitalized patients. In a study of databases reporting side effects of anti‐cholinergics, Salahudeen *et* *al*. (2015) compiled a list of those anti‐cholinergics frequently prescribed and indicated that medicated patients suffer more frequent falls and hip fractures, increased dyskinesias, and suffer from hallucinations, blurry vision, and memory impairment than non‐medicated patients.

The elevation of cholinergic signaling in PD is directly related to the alterations in ChI spiking (Tanimura *et* *al.,* 2018). As described before, M_4_ autoreceptors in ChIs slow firing rate and ACh release (Zhang *et* *al.,* 2002b). In the rodent model of PD, dopamine depletion induces an upregulation of RGS4‐dependent processes that result in decreased M_4_ signaling in ChI (Ding *et* *al.,* 2006). Alternative RGS modulation of ACh release might aid future treatment of patients. Experiments using the same animal model of PD report that halorhodopsin photoinhibition of ChIs in mice reduces akinesia, bradykinesia, and sensory motor neglect; however, in wild‐type mice, the specific striatal blockade of M_1_ and M_4_ receptors has a similar effect. This suggests that the main participants in the absence of ACh are likely the M_1_ and M_4_ receptors since specific striatal blockade of M_1_ and M_4_ receptors has a similar effect (Ztaou *et* *al.,* 2016). These results agree with the electrophysiological studies of muscarinic and dopaminergic interactions described in (Hernandez‐Flores *et* *al.,* 2015).

Recently Burbulla *et* *al*. (2017), using long‐term cultures of human‐induced pluripotent stem cells‐derived dopamine neurons, has demonstrated a toxic cascade triggered by dysfunctional mitochondria that can induce neuronal pathological changes and cellular dysfunctions observed in PD. Now, research is centered on whether the same toxic mitochondrial intracellular cascade is present in the genetic and idiopathic forms of the disease. More work may eventually demonstrate the primary cause of SNc dopamine neuron death.

Dystonia involves intermittent or sustained abnormal involuntary muscle contractions that produce twisting postures in the absence of other neurological signs. Repetitive movement and uncontrolled muscle contractions can start early in childhood (Valente *et* *al.,* 1998; Klein & Fahn, 2013). Early onset of dystonia is a genetically determined mutation in the gene TOR1A (Sciamanna *et* *al.,* 2012). As in PD, the reciprocal modulation between dopamine and ACh is at the center of dystonia. For instance, high doses of anti‐cholinergics (trihexyphenidyl) are used in the treatment of this disease (Burke *et* *al.,* 1986). Electrophysiological experiments in ChIs of mice overexpressing mutant torsin A show that the sensitivity of a D_2_ agonist‐mediated inhibition of Ca_v_2.2 N‐type current is increased. Following D_2_ agonists, a reduction in mAHP and threshold for action potentials is expected (Sciamanna *et* *al.,* 2011). In mice with a conditional knockout of the dystonia 1 protein, the activation of thalamostriatal inputs induces a short pause and increased rebound activity in ChIs that could result from a postsynaptic increase and a presynaptic decrease in M_1_ and M_2_‐dependent currents (Sciamanna *et* *al.,* 2012).

Gilles de la Tourette's syndrome is a neurodevelopmental disorder characterized by motor and phonic tics, usually measured by the Yale Global Tic Severity Scale (Leckman *et* *al.,* 1989). In the last few years, several advances have been achieved toward the understanding of the neuropathology of this syndrome.

The participation of ChIs in this syndrome is supported by postmortem findings of a significant 49% loss of cholinergic and 42% loss of parvalbumin‐positive FS interneurons with a no significant change in ≈ 20% in DARPP‐32 expression in MSNs (Kataoka *et* *al.,* 2010); however, targeted toxin lesion of ChIs in the dorsolateral striatum of adult mice fails to show any abnormal stereotypes (Xu *et* *al.,* 2015). Moreover, the radiotracer [^18^F] fluoroethoxy‐benzovesamicol that is successfully used to image overexpressed vAChT in mice (Janickova *et* *al.,* 2017) failed to detect changes in the number of ChIs in Tourette's syndrome patients (Albin *et* *al.,* 2017), perhaps obscured by the pedunculopontine cholinergic afferents.

Since stereotypy is regarded as a predominant aspect of this syndrome, using cocaine‐induced stereotyped behaviors to test the function of ChIs, it is observed that a lesion of ChI or blockade of mAChR (scopolamine) prolongs the time course of the stereotypy, whereas blockade of dopamine D_2_ receptors (raclopride) stops the stereotypy presumably by increasing the extracellular cholinergic concentration (Aliane *et* *al.,* 2011). These results suggest that a restoration of cholinergic transmission may have important consequences in the arrest of stereotypy. This is supported by a decrease in stereotyped behaviors in children following the administration of a cholinesterase inhibitor (donepezil) (Cubo *et* *al.,* 2008).

Pharmacological animal models of the syndrome have been produced following blockade of striatal GABA_A_ receptors. In mice, rats, and monkeys, intrastriatal administration of specific GABA_A_ antagonists (picrotoxin or bicuculine) induces increased activity in striatum and its outputs (i.e., subthalamic nucleus and thalamus) and motor abnormalities similar to tics (McCairn *et* *al.,* 2009; Bronfeld *et* *al.,* 2013), for review, see Yael *et* *al*. (2015).

Huntington's is a progressive late‐onset neurodegenerative disease characterized by psychiatric symptoms and cognitive deficit. It is caused by a CAG trinucleotide repeat in the gene encoding huntingtin. The resulting huntingtin accumulates forming inclusion bodies with other proteins, initially in neurons of striatal and cortical motor and prefrontal areas (Shepherd, 2013). In postmortem human tissue and rodent models of the disease, there is a striatal pre‐ and postsynaptic loss of GABA, glutamate, dopamine, and muscarinic acetylcholine receptors (Penney & Young, 1982; Dure *et* *al.,* 1991) and a preferential degeneration of MSNs (Reiner *et* *al.,* 1988) with a faster loss in iMSNs (Cha *et* *al.,* 1998; Deng *et* *al.,* 2004; Starr *et* *al.,* 2008). Although the number of ChIs is relatively normal (Ferrante *et* *al.,* 1987), these interneurons have decreased the levels of vAChT and ChAT (Smith *et* *al.,* 2006). In an animal model of the disease (Q140 huntington‐like mice), Deng & Reiner (2016) studied the specific vGLUT2 thalamic inputs to ChIs. They observed a reduction in the extension of the dendritic trees, with a subsequent loss of synapses, as also reported before (Deng *et* *al.,* 2013). The authors propose that a reduced thalamic excitatory drive onto iMSNs could be responsible for an initial observed hyperkinesia in mice. Then, a subsequent loss of dMSNs could lead to the permanent hypokinesia in this animal model.

In recent years, interest has shifted in somewhat different directions. Two examples: (i) attention to the posttranslational modifications of huntingtin by the covalent attachment of a small ubiquitin modifier (SUMO) protein (PIAS1). PIAS1 participates in the huntingtin accumulation of inclusion bodies and as expected, a reduction in PIAS1 prevents the formation of inclusion bodies and reduces inflammation (Ochaba *et* *al.,* 2016). (ii) Attention to the participation of NMDA receptors in neuronal degeneration pointing to the molecular link between mutant huntingtin and the synaptic retrieval of the GluN3A subunit of the NMDA receptors. Mutant huntingtin redirects an intracellular store of juvenile NMDA+GluN3A to the surface of the neurons favoring neuronal loss. Overexpression of GluN3A in normal mice induced synapse loss. Moreover, as expected, the genetic ablation of GluN3A subunits improves motor performance and decreases cell loss in mutant mice (Marco *et* *al.,* 2013).

## Conclusions and future directions

There is an emerging idea that like dopamine, ACh is necessary at a minimum concentration to maintain striatal function. The complex distribution of the receptors for ACh and the tonic activity in the cells themselves suggests a ‘maintenance’ role. The input to these interneurons from cortex and thalamus allows them access to goal‐directed behavioral contexts (from cortex?) and to attentional and arousal internal signals (from thalamus?). The pause in firing that accompanies newly learned cues is similar in timing with the burst of dopamine activity that itself may generate the later burst of activity in the ChIs.

It is easy to imagine that these temporary changes in extracellular transmitter concentrations are a mechanism to remodel striatal functional microcircuits to adjust to the change in circumstances that initiated the pause. The intimate involvement of ACh in the long‐term changes in excitability in striatal cells *in vitro* is also an indication that such a scheme might be involved in the response to novel cues that are recognized as significant by the animal. In this scenario, the distribution of receptors on both cells and terminals suggests that the organization of synaptic microcircuits in the striatum might underlie the changes in functional assemblies that result in changes in behavior.

Methods to identify these functional assemblies and demonstrate their sensitivity to local transmitter concentrations are being developed. They will provide information about the detailed physiology of such changes in function and perhaps begin to make sense of the detailed receptor localizations in the striatal microcircuitry. Work on optogenetic manipulation of the ‘maintenance transmitters’ is already leading to direct tests of their role. Moreover, methods to image activity, at single cell resolution, in groups of related neurons in freely moving animals are developing. We are reaching a time when such ideas cease to be speculation and become testable hypotheses about the role of acetylcholine in animal behavior.

## Conflict of interest

The authors declare no conflict of interest, financial, or otherwise.

## Author contributions

N.A. and T.H.F. wrote the manuscript; M.G.M. wrote some sections and edited the manuscript; T.H.F. made the figures. G.W.A. reviewed the manuscript and provided formulation of comprehensive research goals, mentorship, and leadership.
