# Supplementary material for: Cholinergic modulation of striatal microcircuits
Source: Eur J Neurosci. 2018 Nov 29;49(5):604–22. doi: 10.1111/ejn.13949 (PMC6587740; doi:10.1111/ejn.13949)
Supplement: Supplementary file 1 [file EJN-49-604-s001.pdf]

EJN European Journal of Neuroscience
FENS Federation of European Neuroscience Societies

Cholinergic modulation of striatal microcircuits

Nilupaer Abudukeyoumu, Teresa Hernandez-Flores, Marianela Garcia-Munoz and Gordon W. Arbuthnott

---

Review timeline:	Submission date:	30 November 2017
	Editorial Decision:	08 January 2018
	Revision Received:	14 February 2018
	Editorial Decision:	05 March 2018
	Revision Received:	30 March 2018
	Accepted:	04 April 2018

Editor: Paul Bolam

1st Editorial Decision 08 January 2018

Dear Gordon,

Your manuscript has been reviewed by three external reviewers as well as by the editorial team. We are pleased to say that it is likely to be accepted for publication in the Special Issue of EJN after a few revisions.

As you can see, the reviewers like your review but they all raise some points that need to be addressed. Reviewers 2 and 3 raise issues that can be dealt with relatively easily, however reviewer 1 raises issues that need a little more thought and effort. Please address each of their points carefully in the revised version.

In addition we noted a few points, listed below, that need to be addessed. And, Gordon, you once told me never to put a table of references in a review as it very easy to check whether all the appropriate papers are referenced. As the field is so close to my heart I went through it in detail and note that many of the older papers are missing! I list just a few below (which happen to be mine!). Please check it carefully!

Izzo, P.N. and Bolam, J.P. (1988) Cholinergic synaptic input to different parts of spiny striatonigral ....
Bennett, B.D. and Bolam, J.P. (1994) Synaptic input and output of parvalbumin-immunoreactive neuron.........
Reference 6 in fig: Somogyi, Bolam Smith 1981.
Refrence 8 in fig:Dube et al
Huerta-Ocampo, I., Mena-Segovia, J. and Bolam, J.P. (2014). Convergence of cortical and thalamic in......
Reference 14: Freund et al 198x

Editorial points:
- English needs some attention:
e.g Abstract
'interaction evidenced with the'
'effects of inputs to and from cholinergic interneurons'
- Abstract needs to better summarize the review
- Figures should not be embedded in the text
- An abbreviation list needed

Thank you for submitting your work to EJN.

Kind wishes,

Paul & John
co-Editors in Chief, EJN

Reviews:

Reviewer: 1 Paul Apicella (CNRS, Aix Marseille Université, France)

Comments to the Author

This is yet another contribution to the surge of interest in the organization and function of the local cholinergic innervation of the striatum. However, it remains difficult to say how the present manuscript differs from other recent reviews in the topic area (Deffains and Bergman 2015; Apicella 2017, Yamanaka et al. 2017, J Neural Transm; Tanimura et al. 2017, Eur J Neurosci; Zhang and Cragg 2017, Front Syst Neurosci). My general impression is that the authors have not make a thorough evaluation of the available literature in order to figure out how their review can help to bring a better understanding of the role of ACh in the striatum. They keep a comprehensive inventory of the related work and its results, but without any new insight or attempt to a synthesis. Another troublesome thing is the lack of Figures (only a single one) that makes the manuscript a little dry. If possible, the authors should make greater efforts to conceive additional illustrations that would render the topic more attractive to the readership. If we look at the text in detail, there are some difficulties and inconsistencies that need to be addressed, as discussed below.

Abstract
- « The purpose of this paper… » : The authors should clearly state from the outset that it is a literature review
- « the striatal microcircuit / striatal microcircuits ». In several places in the text, the authors used either the singular or the plural form. It would be desirable to use a coherent wording for this.
- « the selective presence of these interneurons in the striosome and matrix compartments » Where does the selectivity lie ? I mean, in which striatal compartment ? ChIs appear to be located preferentially between matrix and striosomes. Is it the selectivity to which the authors refer ?
- « A general view of the effect of acetylcholine in the striatal microcircuit is the main aim of this review ». Unfortunately, as mentioned before, it is hard to see such a general view in the present text.

Figure
In the Figure, it is unfortunate that MSNs are presented graphically quite similar to interneurons. Wouldn't it be possible to highlight those output neurons so that the reader may better understand that local interactions occur via their axon collaterals? Also the single DA input on ChIs is misleading, unless you specify that you have chosen to illustrate only the DA-ChI connection for clarity.

General remarks
- « ACh » is correctly written with the capital letter C most of the time. Please, check spelling in the text (« Ach » lines 271, 355 and perhaps elsewhere).
- The authors seem to suggest that the tonically active firing is a characteristic of ChIs, but in other parts they argue - I think adequately - that ChIs « have similar firing properties to some GABAergic interneurons that can cause some confusion ». This point needs to be clarified once and for all so that the « tonic firing » alone cannot be used as a criterion to make an absolutely firm conclusion about the cholinergic nature of striatal neurons.
- Frankly, the last paragraph (Conclusion and Future Directions) is not very appealing. What do the authors mean by « the systematic approach to interneuron research » ? It is a bit odd to me.

Text
Line 63. « (dMSN and iMSN), respectively » the 2nd bracket is incorrectly placed.

Lines 103-106. The authors report with the utmost precision that « an individual ChI forms an average 752 ± 62 synapses with vGluT2-positive terminals (Doig et al., 2014). This indicates that ChI receive a prominent inhibitory input and most of its excitatory input is from thalamic afferents ». This statement is important if one compares it with the number of synapses with cortical terminals which is given in the Doig et al.'s paper, suggesting that ChIs form more synapses with thalamic than cortical terminals. The mean number of thalamic synapses in itself does not highlight this fact.

Lines 144-145. « TANs were confirmed as interneurons when antidromic stimulation from globus pallidus was unable to activate them ». I have a little bit of an issue with this preemptory assertion. It has long been known that identification of MSNs by antidromic activation (i.e., pallidal or nigral stimulation) is not reliable. So I do not think that this criterion may allow for the proper identification of striatal interneurons.

Lines 162-163. « Axon collaterals of MSN, contact ChI (Bolam et al., 1986; Lapper & Bolam, 1992; Bennett & Wilson, 1998; Gonzales et al., 2013; Guo et al., 2015), particularly iMSNs (Gonzales et al., 2013) ». This latter statement is obviously wrong. Although the degree of target selectivity of MSN collaterals within the striatal circuitry remains to be further characterized, previous studies in rodents have provided evidence that dMSNs – rather than iMSNs - form synapses with ChIs (Bolam et al. 1986; Chang and Kita 1992; Martone et al. 1992), suggesting specific interactions between ChIs and dMSNs. On the other hand, in monkeys, ChIs receive inputs from axon collaterals of both dMSNs and iMSNs (Gonzales et al. 2013) emphasizing differences between species.

Lines 166-167. « Some interactions of ChIs with other interneurons occur with the GABAergic subtype NPY-low threshold spiking subtype are reciprocally connected (Vuillet et al., 1992), and also between ChIs » Can you add a reference for ChI-ChI interactions mentioned ?

Lines 181-182. « GABAergic axons from GP form a striatal afferent system that connects ChIs among other cells ». This comment is puzzling at best. I'm not aware of what the authors are referring to. It is absolutely necessary to clarify it, given the growing interest in the connectivity and function of the pallido-striatal connection.

Line 182. « There are two neuronal populations in GP, type A (GP-TA) and the GP prototypic (GP-TI) » The meaning of the abbreviation « A » is given a bit later (arkypallidal neurons). Better to give it immediately after the first introduction of the abbreviation.

Lines 188-189. « Evidence of arkypallidal afferents to striatum are involved in stopping behavior but the involvement of individual striatal neuronclasses (add a space) is yet to be described (Mallet et al., 2016) ». This sentence is poorly formulated. I do not know precisely the point to which the authors are referring about « striatal neuron classes » and GP afferents.

Lines 293-294. « the possible discovery of new interneuron types that also interact with ChIs, might result in a complex striatal microcircuit dynamic raising new questions about their physiological role and pathological relevance » Maybe it's true, but I cannot see any way in which this comment provides us with any more clarity in the current status of the issue.

Lines 347-351. In the paragraph dealing with ACh/Glu co-release, subparagraphs are poorly labelled which discomforts reading. May be you can use a new line at the end of each subpart to better emphasize the A and B strategies ? Also, the last sentence (i.e., « No doubt more and surprising data are being collected ») seems to come out of nowhere. What are you thinking about ? computational network models of MSNs and ChIs ? As it is written, the text may give the impression that the vGluT3 is present in different striatal neuron types, including ChIs. Can you be more specific ?

Line 364. « Almost 40 years ago (Graybiel & Ragsdale, 1978) reported… » remove parentheses (Graybiel & Ragsdale (1978))

Line 385. I find the headings confusing « Influence of ChI on striatal plasticity of Medium spiny neurons ». A little further, we find « Cholinergic interneurons » and « GABAergic interneurons », but it is not obvious that the authors refer to the influence of ChIs on plasticity of distinct components of the striatal circuitry. Also, the wording is awkward : « striatal plasticity of » MSNs, ChIs, etc. seems to me inappropriate.

Lines 440-441. « Close to one hundred active neurons are necessary… » Where did that number come from? Also, what do you mean by «… for the mathematical analyses to reveal interacting microcircuits » ? Which studies are you referring to ? Computational network modeling of MSNs and ChIs ? If this is the case, references would be welcome.

Lines 562-563. « The interrelation ACh-dopamine and ACh-GABA suggests their direct involvement in the sculpting the functional microcircuits involved in behavior » This sentence seems incorrect to me. Please rephrase.

Reviewer: 2 Jose Bargas (ENAM, Mexico)

Comments to the Author
EJN-2017-11-25136(IBAGS)

GENERAL
A timely and exhaustive review about CHINs role in the striatal microcircuit. Appropriate for the readers of the European Journal of Neuroscience. It is going to be a reference review with a high impact in the Basal Ganglia community.
My observations are opinions, minor and conceptual, more to facilitate heuristic questioning than to be mandatory for the authors who already made a great job.
They also take advantage of the authors experience working at three different levels: cellular, microcircuits and systems/behavioral.

Examples of comments that need to have some more explanation from the authors:
Lines 105-106 ("…most of its excitatory input is from thalamic afferents.") have no references and appear to contradict lines 207-208 ("… optogenetic stimulation of striatal glutamatergic afferents from cortex or thalamus increase spike probability and firing rate in ChIs…") and 317-325 ("Activation of presynaptic M2 and M3 receptors on cortical striatal afferents…decreases glutamate release with a resulting…decrease of MSN glutamate-dependent excitatory drive…").

On the other hand, lines talking about different locations of contacts on the dendrites (lines 196-197) do not say anything about their electrotonic influence at the soma where action potentials are generated. No matter the anatomical location, physiologically, the important point is their influence to generate action potentials. The lines above suggest that both cortex and thalamus may do that.

As the authors themselves mention, there is still confusion. Therefore, caution and careful signaling of this lack of congruency may help guide future research. This reviewer thinks that the functional preferred innervation of cortical or thalamic afferents on ChIs or on indirect pathway projection neurons is in debate, until further experimental evidence is gathered.

In the same token, lines 209-217: what is the combined effect of some mGluRs increasing postsynaptic excitability and some other mGluRs decreasing glutamate release presynaptically from the same afferents that cause the excitation? I guess, these apparent contradictions cannot be solved at the cellular level, but perhaps have an answer by studying the microcircuit level, which is suggested in the Conclusions but not in the microcircuits section. These points need to be explicitly indicated as they are: apparent contradictions, otherwise their importance is underestimated. Apparent contradictions since the actions are there for some physiological reason. Sometimes papers are rejected because reviewers study one of the actions and not the opposite (!), a main reason to remark them.

In sum, apparent inconsistencies at the cell level may not be so at the circuit level and since the review title is intended to shed light on microcircuits actions, this reviewer asks for some ideas/speculations about these apparent contradictions.

A similar point can be raised about CHIs expressing D2 and D1/5 receptors, although here, differences in sensitivity may suggest easy hypotheses, there is one problem: how L-DOPA administration may change them.

The review is full of examples like these: presynaptic cholinergic receptors in glutamatergic afferents, some inhibit, others increase release. Why the terminal needs that?

As the authors say in line 361: "…surprising data…", but the readers may be helped by pointing them out more explicitly.

In any case the review is full of useful information and has a very complete set of references. My suggestions above are a little bit mentioned under the section of "Conclusions and future directions".

Minor suggestions:
Line 49: it should say "ligand gated ion channels".

In the Parkinson´s section this reference may be cited:
Tanimura et al. 2017 EJN doi:10.1111/ejn.13638

In the Tourette section some more references are perhaps needed:

Bronfeld M, Yael D, Belelovsky K, Bar-Gad I. 2013 Motor tics evoked by striatal disinhibition in the rat. Front Syst Neurosci 7:50. doi: 10.3389/fnsys.2013.00050

Yael D, Vinner E, Bar-Gad I. (2015) Pathophysiology of tic disorders. Mov Disord 30: 1171-1178. doi: 10.1002/mds.26304.

Reviewer: 3 Margaret Rice (NYU School of Medicine, USA)

Comments to the Author
This is a timely and well-written review of the role of cholinergic interneurons (ChIs) in striatal function. The review provides more breadth than depth of coverage for specific topics (like ionic conductances of ChIs), but this fills a useful gap in the literature. The concerns below include suggestions to improve clarity and consistency, all of which are minor.

39. This section heading misses the main focus of the section on cholinergic receptors.

58-66. The logic of presenting mAChRs in order of their numbering scheme makes sense, but somehow diminishes their role of as key ChI autoreceptors. By contrast, in the nAChR presentation just before this section, nAChR autoreceptors on ChIs are mentioned first, over-amplifying their role.

99-106. For this general description, "23-50 µm" would be better; next measurement should be 1 mm. If means are given for synaptic contacts, SD should be indicated, with n values given if SEM. Also, these precise (and large) numbers of synaptic contacts seem at odds with the statement on the previous page that ChI axons "form few structurally defined synaptic connections". Certainly this says afferent synapses, but perhaps li. 102-103 could say "receives" (or similar) instead of "has" and "forms" to avoid confusion. Perhaps the last sentence of the paragraph could precede the quantitative data.

134. The intrinsic properties "allow ChIs to fire…".

271, 341, 355. ACh

300. "This evidence…"

303-312. The work of Descarries et al. (1996, 1997) suggests few cholinergic synapses on DA axons; this section should be reconsidered in this light. Indeed, conventional wisdom is that potent cholinergic regulation of DA release is by volume transmission (e.g., Threlfell and Cragg, 2011).

346. "Co-release from ChIs"?

353-361. The use of A- and B- separated by several sentences is cumbersome. This section is important, but needs to be revised to be clear.

369. Remove comma.

382. Better might be to say, "reaffirm the location of ChIs between, as well as within, matrix and striosome…".

468-473. In considering ACh-dopamine interactions, it might be worth noting a behavioral consequence of genetic loss of striatal ACh synthesis is hyperactivity in a novel environment (Patel et al., 2012).

470, 475. Need PD definition only once.

475-485, 502. It would be helpful to mention the class(es) anticholinergic medications used therapeutically.

483. Needs parallel structure to compare side effects seen in patients who are on anticholinergics to those who are not.

489-494. Inhibiting ChIs with halorhodopsin would decrease release of ACh acting at all receptors, whereas antagonizing M2 and M4 receptors

would be expected to have the opposite effect, as autoreceptor blockade should increase ACh release. This needs to be clarified.

Authors' Response 14 February 2018

Dear Paul,

Here it is at last. I hope you like it better.

We were challenged but encouraged by the reviews and we think we have a very much better paper because of their insightful comments. We hope this is more to their liking, we like it better too. We hereby submit the manuscript "Cholinergic modulation of striatal microcircuits" which is a resubmission for publication in European Journal of Neuroscience.

Looking forward to seeing it in the IBAGS issue.

Best Wishes,
Gordon.

Here we address the general, editorial and specific points raised by the reviewers.

General and editorial points-
Gordon, you once told me never to put a table of references in a review as it very easy to check whether all the appropriate papers are referenced. As the field is so close to my heart I went through it in detail and note that many of the older papers are missing! I list just a few below (which happen to be mine!). Please check it carefully!
Reply- More references have been included in the table including the ones listed in lines 352, 405, and Table 1 rows: 2, 6, 8, 13 14, 16. Besides the following note was added at the end of Table 1 and Table 2: "Note: These selected references by no means reflect all the evidence gathered through more than 40 years of research, apologies for unintended omissions."

English was corrected √
Abstract needs to better summarize the review √
Embedded figure removed √
Abbreviation list included separately √

Reviewer: 1
General
Comment-This is yet another contribution to the surge of interest in the organization and function of the local cholinergic innervation of the striatum. However, it remains difficult to say how the present manuscript differs from other recent reviews in the topic area (Deffains and Bergman 2015; Apicella 2017, Yamanaka et al. 2017, J Neural Transm; Tanimura et al. 2017, Eur J Neurosci; Zhang and Cragg 2017, Front Syst Neurosci). My general impression is that the authors have not make a thorough evaluation of the available literature in order to figure out how their review can help to bring a better understanding of the role of ACh in the striatum. They keep a comprehensive inventory of the related work and its results, but without any new insight or attempt to a synthesis.
Reply- We believe our review contributes information from a detached point of view. Recent reviews include references and illustrations of data often obtained by the authors of the review. This approach likely unintentionally can incline the reader towards a specific conclusion. We think that our approach provides the reader with the opportunity to conceive new ideas. However, attempts were made throughout and the conclusions also attempt a synthesis to provide insight.

Comment- Another troublesome thing is the lack of Figures (only a single one) that makes the manuscript a little dry. If possible, the authors should make greater efforts to conceive additional illustrations that would render the topic more attractive to the readership.
Reply- Now the manuscript has three illustrations

Difficulties and inconsistencies
Comment-Abstract. Clearly state from the outset that it is a literature review
Reply- A new abstract was written that clearly states in the first sentence that it is a literature review.

Comment-the striatal microcircuit / striatal microcircuits ». In several places in the text, the authors used either the singular or the plural form. It would be desirable to use a coherent wording for this.
Reply- Corrected to "striatal microcircuits".

Comment-«the selective presence of these interneurons in the striosome and matrix compartments » Where does the selectivity lie? I

mean, in which striatal compartment? ChIs appear to be located preferentially between matrix and striosomes. Is it the selectivity to which the authors refer?
Reply- This sentence is from the abstract. We devote a whole section (lines 559-590) to the topic. The abstract was rewritten.

Comment- « A general view of the effect of acetylcholine in the striatal microcircuit is the main aim of this review ». Unfortunately, as mentioned before, it is hard to see such a general view in the present text.
Reply- This sentence is from the abstract. The abstract was rewritten and the original section "ACh and striatal microcircuits" is still there, plus apart from title, some subtitles and title and legend of Figure 1, more comments were inserted to remind the reader that we are talking about striatal microcircuits (mentioned 18 times) as compared to the first version (mentioned 6 times)

Comment- In the Figure, it is unfortunate that MSNs are presented graphically quite similar to interneurons. Wouldn't it be possible to highlight those output neurons so that the reader may better understand that local interactions occur via their axon collaterals? Also, the single DA input on ChIs is misleading, unless you specify that you have chosen to illustrate only the DA-ChI connection for clarity.
Reply- We shaded the circles of the MSNs to give them a different effect from the rest.
General remarks
« ACh » is correctly written with the capital letter C most of the time. Please, check spelling in the text (« Ach » lines 271, 355 and perhaps elsewhere).
Reply- Corrected

The authors seem to suggest that the tonically active firing is a characteristic of ChIs, but in other parts they argue - I think adequately - that ChIs « have similar firing properties to some GABAergic interneurons that can cause some confusion ». This point needs to be clarified once and for all so that the « tonic firing » alone cannot be used as a criterion to make an absolutely firm conclusion about the cholinergic nature of striatal neurons.c
Reply- Corrected. Characteristics of cholinergic interneurons: Electrophysiological. Last paragraph

Comment- Frankly, the last paragraph (Conclusion and Future Directions) is not very appealing. What do the authors mean by « the systematic approach to interneuron research »? It is a bit odd to me.
Reply-Conclusion rewritten

Comment- Line 63. « (dMSN and iMSN), respectively » the 2nd bracket is incorrectly placed.
Reply- Corrected to "striatal MSNs of both the direct -dMSN- and indirect -iMSN- pathways."

Comment- Lines 103-106. The authors report with the utmost precision that « an individual ChI forms an average 752 ± 62 synapses with vGluT2-positive terminals (Doig et al., 2014). This indicates that ChI receive a prominent inhibitory input and most of its excitatory input is from thalamic afferents ». This statement is important if one compares it with the number of synapses with cortical terminals which is given in the Doig et al.'s paper, suggesting that ChIs form more synapses with thalamic than cortical terminals. The mean number of thalamic synapses in itself does not highlight this fact.
Reply- paragraph Characteristics of cholinergic interneurons:Anatomical was revised.

Comment- Lines 144-145. « TANs were confirmed as interneurons when antidromic stimulation from globus pallidus was unable to activate them ». I have a little bit of an issue with this preemptory assertion. It has long been known that identification of MSNs by antidromic activation (i.e., pallidal or nigral stimulation) is not reliable. So I do not think that this criterion may allow for the proper identification of striatal interneurons.
Reply- the descriptions now reads: "Following electrophysiological criteria, TANs were considered as putative ChIs when antidromic stimulation from globus pallidus (GP) was unable to activate them (Kimura et al., 1990; Kimura et al., 1996). Moreover, in view of their morphological, electrophysiological, regional, functional and immunoreactivity similarities, TANs were identified as ChIs …"

Comment- Lines 162-163. « Axon collaterals of MSN, contact ChI (Bolam et al., 1986; Lapper & Bolam, 1992; Bennett & Wilson, 1998; Gonzales et al., 2013; Guo et al., 2015), particularly iMSNs (Gonzales et al., 2013) ». This latter statement is obviously wrong. Although the degree of target selectivity of MSN collaterals within the striatal circuitry remains to be further characterized, previous studies in rodents have provided evidence that dMSNs – rather than iMSNs - form synapses with ChIs (Bolam et al. 1986; Chang and Kita 1992; Martone et al. 1992), suggesting specific interactions between ChIs and dMSNs. On the other hand, in monkeys, ChIs receive inputs from axon collaterals of both dMSNs and iMSNs (Gonzales et al. 2013) emphasizing differences between species.
Reply- References were added to Characteristics of cholinergic interneurons:Anatomical and paragraph was corrected.

Comment- Lines 166-167. « Some interactions of ChIs with other interneurons occur with the GABAergic subtype NPY-low threshold spiking subtype are reciprocally connected (Vuillet et al., 1992), and also between ChIs » Can you add a reference for ChI-ChI interactions mentioned?
Reply- in Characteristics of cholinergic interneurons:Anatomical reference (Pakhotin & Bracci, 2007) was added in this paragraph and in Table 1.

Comment- Lines 181-182. « GABAergic axons from GP form a striatal afferent system that connects ChIs among other cells ». This

comment is puzzling at best. I'm not aware of what the authors are referring to. It is absolutely necessary to clarify it, given the growing interest in the connectivity and function of the pallido-striatal connection.
Reply- Removed

Comment- Line 182. « There are two neuronal populations in GP, type A (GP-TA) and the GP prototypic (GP-TI) » The meaning of the abbreviation « A » is given a bit later (arkypallidal neurons). Better to give it immediately after the first introduction of the abbreviation.
Reply- Details of the GP-inputs were detailed in first paragraph of Extrastriatal:GABAergic

Comment- Lines 188-189. « Evidence of arkypallidal afferents to striatum are involved in stopping behavior but the involvement of individual striatal neuronclasses (add a space) is yet to be described (Mallet et al., 2016) ». This sentence is poorly formulated. I do not know precisely the point to which the authors are referring about « striatal neuron classes » and GP afferents.
Reply- paragraph Extrastriatal:GABAergic was rewritten

Comment- Lines 293-294. « the possible discovery of new interneuron types that also interact with ChIs, might result in a complex striatal microcircuit dynamic raising new questions about their physiological role and pathological relevance » Maybe it's true, but I cannot see any way in which this comment provides us with any more clarity in the current status of the issue.
Reply- Sentence deleted

Comment- Lines 347-351. In the paragraph dealing with ACh/Glu co-release, subparagraphs are poorly labelled which discomforts reading. May be you can use a new line at the end of each subpart to better emphasize the A and B strategies ? Also, the last sentence (i.e., « No doubt more and surprising data are being collected ») seems to come out of nowhere. What are you thinking about ? computational network models of MSNs and ChIs ? As it is written, the text may give the impression that the vGluT3 is present in different striatal neuron types, including ChIs. Can you be more specific?
Reply- The paragraph entitled “Co-release from ChIs” has been re-written.

Comment- Line 364. « Almost 40 years ago (Graybiel & Ragsdale, 1978) reported… » remove parentheses (Graybiel & Ragsdale (1978))
Reply- Corrected

Comment- Line 385. I find the headings confusing « Influence of ChI on striatal plasticity of Medium spiny neurons ». A little further, we find « Cholinergic interneurons » and « GABAergic interneurons », but it is not obvious that the authors refer to the influence of ChIs on plasticity of distinct components of the striatal circuitry. Also, the wording is awkward : « striatal plasticity of» MSNs, ChIs, etc. seems to me inappropriate.
Reply- This section Participation of cholinergic interneurons in striatal plasticity has been rewritten and old subtitles removed.

Comment- Lines 440-441. « Close to one hundred active neurons are necessary… » Where did that number come from? Also, what do you mean by «… for the mathematical analyses to reveal interacting microcircuits »? Which studies are you referring to ? Computational network modeling of MSNs and ChIs? If this is the case, references would be welcome.
Reply- The first paragraph in section ACh and the striatal microcircuits has been corrected and references added.

Comment- Lines 562-563. « The interrelation ACh-dopamine and ACh-GABA suggests their direct involvement in the sculpting the functional microcircuits involved in behavior » This sentence seems incorrect to me. Please rephrase.
Reply- Conclusion has been rewritten.

Reviewer: 2
General
A timely and exhaustive review about CHINs role in the striatal microcircuit. Appropriate for the readers of the European Journal of Neuroscience. It is going to be a reference review with a high impact in the Basal Ganglia community.
My observations are opinions, minor and conceptual, more to facilitate heuristic questioning than to be mandatory for the authors who already made a great job.
They also take advantage of the authors experience working at three different levels: cellular, microcircuits and systems/behavioral.

Examples of comments that need to have some more explanation from the authors:
Comment- Lines 105-106 (“…most of its excitatory input is from thalamic afferents.”) have no references and appear to contradict lines 207-208 (“… optogenetic stimulation of striatal glutamatergic afferents from cortex or thalamus increase spike probability and firing rate in ChIs…”) and 317-325 (“Activation of presynaptic M2 and M3 receptors on cortical striatal afferents…decreases glutamate release with a resulting…decrease of MSN glutamate-dependent excitatory drive…”).
Reply- Modifications were made as follows:
Lines 105-106: First paragraph of Characteristics of cholinergic interneurons: Anatomical has been modified
Lines 207-208: The section “Afferents to cholinergic interneurons: glutamatergic” has been modified.
Lines 317-325: We address this issue in Figure 3.

Comment- On the other hand, lines talking about different locations of contacts on the dendrites (lines 196-197) do not say anything

about their electrotonic influence at the soma where action potentials are generated. No matter the anatomical location, physiologically, the important point is their influence to generate action potentials. The lines above suggest that both cortex and thalamus may do that.
Reply- The section "Afferents to cholinergic interneurons: glutamatergic" has been modified.

Comment- As the authors themselves mention, there is still confusion. Therefore, caution and careful signaling of this lack of congruency may help guide future research. This reviewer thinks that the functional preferred innervation of cortical or thalamic afferents on ChIs or on indirect pathway projection neurons is in debate, until further experimental evidence is gathered.
Reply- We address this topic section "Influence of cholinergic interneurons within striatal microcircuits: Glutamatergic terminals" Figure 3.

Comment- In the same token, lines 209-217: what is the combined effect of some mGluRs increasing postsynaptic excitability and some other mGluRs decreasing glutamate release presynaptically from the same afferents that cause the excitation? I guess, these apparent contradictions cannot be solved at the cellular level, but perhaps have an answer by studying the microcircuit level, which is suggested in the Conclusions but not in the microcircuits section. These points need to be explicitly indicated as they are: apparent contradictions, otherwise their importance is underestimated. Apparent contradictions since the actions are there for some physiological reason. Sometimes papers are rejected because reviewers study one of the actions and not the opposite (!), a main reason to remark them.
Reply- The section "Afferents to cholinergic interneurons… Glutamatergic" was modified.

Comment- In sum, apparent inconsistencies at the cell level may not be so at the circuit level and since the review title is intended to shed light on microcircuits actions, this reviewer asks for some ideas/speculations about these apparent contradictions.
A similar point can be raised about CHIs expressing D2 and D1/5 receptors, although here, differences in sensitivity may suggest easy hypotheses, there is one problem: how L-DOPA administration may change them.
Reply- The paragraph "Afferents to cholinergic interneurons…Dopaminergic" was modified.

Comment- The review is full of examples like these: presynaptic cholinergic receptors in glutamatergic afferents, some inhibit, others increase release. Why the terminal needs that?
Reply- We attempted to address this question in Figure 3 Legend.

Comment- As the authors say in line 361: "…surprising data…", but the readers may be helped by pointing them out more explicitly.
Reply- Last sentences of the section "Co-release from cholinergic interneurons" suggests some interesting questions.

Comment- In any case the review is full of useful information and has a very complete set of references. My suggestions above are a little bit mentioned under the section of "Conclusions and future directions".

Minor suggestions:
Line 49: it should say "ligand gated ion channels" √ Modified in line 91
In the Parkinson´s section this reference may be cited:
Tanimura et al. 2017 EJN doi:10.1111/ ejn.13638 √Included
In the Tourette section some more references are perhaps needed:
Bronfeld M, Yael D, Belelovsky K, Bar-Gad I. 2013 Motor tics evoked by striatal disinhibition in the rat. Front Syst Neurosci 7:50. doi: 10.3389/fnsys.2013.00050
Yael D, Vinner E, Bar-Gad I. (2015) Pathophysiology of tic disorders.
Mov Disord 30: 1171-1178. doi: 10.1002/mds.26304.
Reply- Included the following references:
McCairn, K.W., Bronfeld, M., Belelovsky, K. & Bar-Gad, I. (2009) The neurophysiological correlates of motor tics following focal striatal disinhibition. Brain, 132, 2125-2138.
Bronfeld, M., Yael, D., Belelovsky, K. & Bar-Gad, I. (2013) Motor tics evoked by striatal disinhibition in the rat. Frontiers in systems neuroscience, 7, 50.
Yael, D., Vinner, E. & Bar-Gad, I. (2015) Pathophysiology of tic disorders. Mov Disord, 30, 1171-1178.

Reviewer: 3
This is a timely and well-written review of the role of cholinergic interneurons (ChIs) in striatal function. The review provides more breadth than depth of coverage for specific topics (like ionic conductances of ChIs), but this fills a useful gap in the literature. The concerns below include suggestions to improve clarity and consistency, all of which are minor.

Comment- 39. This section heading misses the main focus of the section on cholinergic receptors.
Reply- The subtitle was changed to "Striatal acetylcholine receptors"

Comment- 58-66. The logic of presenting mAChRs in order of their numbering scheme makes sense, but somehow diminishes their

role of as key ChI autoreceptors. By contrast, in the nAChR presentation just before this section, nAChR autoreceptors on ChIs are mentioned first, over-amplifying their role.
Reply- We changed them round

Comment- 99-106. For this general description, “23-50 µm” would be better; next measurement should be 1 mm. If means are given for synaptic contacts, SD should be indicated, with n values given if SEM. Also, these precise (and large) numbers of synaptic contacts seem at odds with the statement on the previous page that ChI axons “form few structurally defined synaptic connections”.
Certainly this says afferent synapses, but perhaps li. 102-103 could say “receives” (or similar) instead of “has” and “forms” to avoid confusion. Perhaps the last sentence of the paragraph could precede the quantitative data.
Replies-
99-106 •22.8µm was changed to 23µm
•The space in 1mm was added: 1 mm.
Comment- If means are given for synaptic contacts, SD should be indicated, with n values given if SEM.
Reply- •We left the values as reported by the authors: J.Neurosci. 34(8) page 3107, second paragraph.
102-103 • The word ‘receive’ was included
•The paragraph was rewritten

Comments-
134. The intrinsic properties “allow ChIs to fire…”.
271, 341, 355. Ach
300. “This evidence…”
Reply- All these points were corrected.

303-312. The work of Descarries et al. (1996, 1997) suggests few cholinergic synapses on DA axons; this section should be reconsidered in this light. Indeed, conventional wisdom is that potent cholinergic regulation of DA release is by volume transmission (e.g., Threlfell and Cragg, 2011).
Reply- In the section called “Dopaminergic terminals” the first two paragraphs were changed to address this concern.

346. “Co-release from ChIs”? Reply- Corrected
353-361. The use of A- and B- separated by several sentences is cumbersome. This section is important, but needs to be revised to be clear. Reply- Corrected
369. Remove comma. Reply- Corrected
382. Better might be to say, “reaffirm the location of ChIs between, as well as within, matrix and striosome…” Reply- Corrected

468-473. In considering ACh-dopamine interactions, it might be worth noting a behavioral consequence of genetic loss of striatal ACh synthesis is hyperactivity in a novel environment
(Patel et al., 2012).
Reply- The section Influence of cholinergic interneurons within striatal microcircuits: dopaminergic paragraph before last was added to address this issue.
nserted a new paragraph, lines 477-482 to address this issue.

470, 475. Need PD definition only once. Reply-Corrected
475-485, 502. It would be helpful to mention the class(es) anticholinergic medications used therapeutically. Reply-Added when mentioned by the authors.
483- Needs parallel structure to compare side effects seen in patients who are on anticholinergics to those who are not.
Reply-Sentence modified in lines to indicate that Salahudeen et al. (2015) had performed a study of databases.

489-494. Inhibiting ChIs with halorhodopsin would decrease release of ACh acting at all receptors, whereas antagonizing M2 and M4 receptors would be expected to have the opposite effect, as autoreceptor blockade should increase ACh release. This needs to be clarified.
Reply- In Movement Disorders PD the paragraph before last clarifies this point.

2nd Editorial Decision 05 March 2018

Dear Gordon,

Your revised manuscript has been re-evaluated by original external reviewers as well as by the Editorial team. We are pleased to inform you that we expect that it will be acceptable for publication in EJN following few further minor revisions. Please address the minor points raised by

Reviewers 1 and 3. Please also supply a graphical abstract. The revised version will not require re-review.

Thank you for your support of this Special Issue of EJN.

Best wishes,

Paul & John
co-Editors in Chief, EJN

Reviews:

Reviewer: 2 Jose Bargas (ENAM, Mexico)

Comments to the Author
The review was improved by following reviewers comments. It is very complete now with the new figuras and additions.

Reviewer: 1 Paul Apicella (CNRS, Aix Marseille Université, France

Comments to the Author
The authors' revisions have answered my questions/comments and made this review stronger and clearer. The addition of two new illustrations makes reading easier and allows a quick overview of knowledge on some crucial aspects.
I only noticed a few small flaws:
p.2 "Muscarinic mAChRs belong to the G-protein coupled receptor (GPCR) family" : "muscarinic" and "mAChR" are redundant. The abbreviation is sufficient.

p.4 " a greater concentration of ChIs in the dorsomedial compared to ventrolateral areas was observed following a stereological reconstruction (Matamales et al., 2016)." Please, specify that this is a mouse's study.

p.15 " When considering microcircuits e.g., composed of glutamate-ACh-dopamine synapses the activation of different cholinergic receptors of different affinities or the complete absence of ACh in knock-out mice, can produce different modulatory effects." The sentence seems incorrect to me. Maybe brackets are missing ?

p.15 "... glutamatergic tone Patel et al. (2012)" Incorrectly placed brackets. Also in different parts of the text, the Figures must be mentioned in brackets (example p.16 / Figure 3)

p.25 "... the distribution of receptors on both, cells and terminals, suggests that... " remove comas.

Reviewer: 3 Margaret Rice (NYU School of Medicine, USA)

Comments to the Author
This revised review article addresses a range of factors that influence the activity of ChIs, their role in striatal microcircuits, and, finally, ChI and AChR dysregulation in disease states. As the authors note in their response to the initial reviews, the topics covered are of general interest, and the article fills a novel role of being written from a fresh perspective of those not directly in the field – at least at the moment. Most of the original concerns have been address by extensive re-writing and the addition of new figures. The criticisms listed below are generally minor; most relate to problems with clarity or grammar. The one substantive concern is that the input to ChIs described in this review was determined primarily in dorsal striatum. This should be clearly stated – at least as a general point before physiological characteristics or thalamic vs. cortical input is described. But it is a relevant point to keep in mind throughout.

1) The second sentence of the abstract and first sentence of the manuscript make an important, yet simple, point that high levels of striatal ACh arise from ChIs. However, as written, both sentences are awkward. Deleting the comma in abstract sentence would help, as would simplification of the text sentence that currently reads, "…connections that contribute to give striatum its place among structures…". Also, "the" before "striatal microcircuits" in the next text sentence should be deleted.

2) p. 5, li. 1-3 and later. "Spontaneously active firing is characteristic of ChIs and ensures basal cholinergic tone…" The next sentence should begin, "These neurons have…" Later in this paragraph, the abbreviations BK and SK should be defined if they are to be used subsequently. Also, should be "A decrease in calcium levels reduces the SK…".

3) p. 5, para. 2. Better would be, "…is a long pause in tonic firing that follows a burst of action potentials."

4) p. 7. The authors state that "Striatal microcircuits are mainly formed by connections between MSNs, interneurons and ChIs." Certainly, there is a microcircuit that is "mainly formed by connections between MSNs, interneurons and ChIs." However, this is not the only microcircuit, and not necessarily the main one, as it excludes striatal input – including dopamine. This might be better refer to "A key intrastriatal microcircuit is formed by…", which would be consistent with this section heading (and later sections, as well).

5) p. 4, p. 8-9, etc. Glutamatergic input to ChIs from thalamus and cortex to ChIs has been characterized primarily in dorsal striatum. As noted in the newly cited paper by Matamales et al (2016), however, this likely to differ depending on striatal subregion, reflected in differing dorsal to

ventral gradients of vGluT1 and vGluT2 (e.g., Wouterlood et al 2012). No comparable studies to those of Doig et al have been reported for ChIs nucleus accumbens. This should be noted in the text, including a statement the data summarized here (and in other sections) were primarily from dorsal striatum. This could be added at the beginning of the overall section on input (e.g., p. 4-10), but also elsewhere. It would also be helpful for the authors to give their perspective on the extent to which the summary of input distribution from dorsal striatum are likely to be similar in ventral striatum (NAc core and shell).

6) p. 15. The new paragraph is confusing; existing evidence indicates that α7-nAChRs show little desensitization, whereas α4β2* desensitize readily with either ACh or nicotine (e.g., Zhou et al. 2001). Also, Patel et al 2012 did not examine glutamatergic regulation, but did show increases in locomotor activity with genetic deletion of forebrain ACh that correlated with increased burst-to-tonic evoked dopamine release throughout the striatum.

7) p. 20. No need for "the" in heading or first sentence (or, in the first sentence, change to "the striatal microcircuits discussed here…".

Authors' Response 30 March 2018

Dear Editors

We are submitting a modified version of this mauscript following the reviewers suggestions.
Thank you very much.
Yours,
Gordon

Manuscript Number:EJN-2017-11-25136(IBAGS).R1

Cholinergic modulation of striatal microcircuits

Comments from- co-Editors in Chief, EJN:
- Please address the minor points raised by Reviewers 1 and 3. Reply- Corrected
- Supply a graphical abstract. Reply-Attached

The revised version will not require re-review.

Reviewer: 1
Comments to the Author
The authors' revisions have answered my questions/comments and made this review stronger and clearer. The addition of two new illustrations makes reading easier and allows a quick overview of knowledge on some crucial aspects.

Comment- I only noticed a few small flaws:
p.2 "Muscarinic mAChRs belong to the G-protein coupled receptor (GPCR) family" : "muscarinic" and "mAChR" are redundant. The abbreviation is sufficient.
Reply- Corrected

Comment- p.4 " a greater concentration of ChIs in the dorsomedial compared to ventrolateral areas was observed following a stereological reconstruction (Matamales et al., 2016)." Please, specify that this is a mouse's study.
Reply- The text now reads: "…however in mice a greater concentration of ChIs in the dorsomedial compared to ventrolateral areas was observed following a stereological reconstruction (Matamales et al., 2016)." p. 4

Comment- p.15 " When considering microcircuits e.g., composed of glutamate-ACh-dopamine synapses the activation of different cholinergic receptors of different affinities or the complete absence of ACh in knock-out mice, can produce different modulatory effects." The sentence seems incorrect to me. Maybe brackets are missing ?
Reply- Corrected, p.14

Comment- p.15 "... glutamatergic tone Patel et al. (2012)" Incorrectly placed brackets.
Reply- Corrected, p.14

Comment- Also in different parts of the text, the Figures must be mentioned in brackets (example p.16 / Figure 3)
Reply- Corrected everywhere.

EJN European Journal of Neuroscience FENS Federation of European Neuroscience Societies

Comment- p.25 "... the distribution of receptors on both, cells and terminals, suggests that... " remove comas.
Reply- corrected

Reviewer: 3
Comments to the Author
This revised review article addresses a range of factors that influence the activity of ChIs, their role in striatal microcircuits, and, finally, ChI and AChR dysregulation in disease states. As the authors note in their response to the initial reviews, the topics covered are of general interest, and the article fills a novel role of being written from a fresh perspective of those not directly in the field – at least at the moment. Most of the original concerns have been address by extensive re-writing and the addition of new figures. The criticisms listed below are generally minor; most relate to problems with clarity or grammar.

Comment- The one substantive concern is that the input to ChIs described in this review was determined primarily in dorsal striatum. This should be clearly stated – at least as a general point before physiological characteristics or thalamic vs. cortical input is described. But it is a relevant point to keep in mind throughout.
Reply- Corrected at the end of the section: "Characteristics of Cholinergic interneurons: Anatomical": "…These afferents, mainly determined in the dorsomedial striatum, are discussed in more detail in a section: "Extrastriatal afferents: glutamatergic". (p.4)

Comment- 1) The second sentence of the abstract and first sentence of the manuscript make an important, yet simple, point that high levels of striatal ACh arise from ChIs. However, as written, both sentences are awkward. Deleting the comma in abstract sentence would help, as would simplification of the text sentence that currently reads, "…connections that contribute to give striatum its place among structures…". Also, "the" before "striatal microcircuits" in the next text sentence should be deleted.
Reply- corrected

Comment- 2) p. 5, li. 1-3 and later. "Spontaneously active firing is characteristic of ChIs and ensures basal cholinergic tone…" The next sentence should begin, "These neurons have…" Later in this paragraph, the abbreviations BK and SK should be defined if they are to be used subsequently. Also, should be "A decrease in calcium levels reduces the SK…".
Reply- corrected, p.5

Comment- 3) p. 5, para. 2. Better would be, "…is a long pause in tonic firing that follows a burst of action potentials."
Reply- corrected, p.5

Comment- 4) p. 7. The authors state that "Striatal microcircuits are mainly formed by connections between MSNs, interneurons and ChIs." Certainly, there is a microcircuit that is "mainly formed by connections between MSNs, interneurons and ChIs." However, this is not the only microcircuit, and not necessarily the main one, as it excludes striatal input – including dopamine. This might be better refer to "A key intrastriatal microcircuit is formed by…", which would be consistent with this section heading (and later sections, as well).
Reply- Corrected

Comment- 5) p. 4, p. 8-9, etc. Glutamatergic input to ChIs from thalamus and cortex to ChIs has been characterized primarily in dorsal striatum. As noted in the newly cited paper by Matamales et al (2016), however, this likely to differ depending on striatal subregion, reflected in differing dorsal to ventral gradients of vGluT1 and vGluT2 (e.g., Wouterlood et al 2012).
No comparable studies to those of Doig et al have been reported for ChIs nucleus accumbens. This should be noted in the text, including a statement the data summarized here (and in other sections) were primarily from dorsal striatum. This could be added at the beginning of the overall section on input (e.g., p. 4-10), but also elsewhere. It would also be helpful for the authors to give their perspective on the extent to which the summary of input distribution from dorsal striatum are likely to be similar in ventral striatum (NAc core and shell).
Reply-Paragraph modified (at the end of page 3, beginning of page 4).

Comment- 6) p. 15. The new paragraph is confusing; existing evidence indicates that α7-nAChRs show little desensitization, whereas $\alpha4\beta2^*$ desensitize readily with either ACh or nicotine (e.g., Zhou et al. 2001). Also, Patel et al 2012 did not examine glutamatergic regulation, but did show increases in locomotor activity with genetic deletion of forebrain ACh that correlated with increased burst-to-tonic evoked dopamine release throughout the striatum.
Reply- Corrected (last and first paragraph, pages 12-13).

Comment- 7) p. 20. No need for "the" in heading or first sentence (or, in the first sentence, change to "the striatal microcircuits discussed here…".
Reply- Corrected (page 17)
